# GADD45B regulates the carcinogenesis process of chronic atrophic gastritis and the metabolic pathways of gastric cancer

**DOI:** 10.3389/fendo.2023.1224832

**Published:** 2023-08-07

**Authors:** Wei Xu, Tianxiao Jiang, Kanger Shen, Dongxu Zhao, Man Zhang, Wenxin Zhu, Yunfei Liu, Chunfang Xu

**Affiliations:** ^1^ Department of Gastroenterology, The First Affiliated Hospital of Soochow University, Suzhou, Jiangsu, China; ^2^ Department of General, Visceral, and Transplant Surgery, Ludwig-Maximilians-University Munich, Munich, Germany; ^3^ Department of Interventional Radiology, The First Affiliated Hospital of Soochow University, Suzhou, Jiangsu, China; ^4^ Department of Emergency Medicine, The Affiliated Hospital of Xuzhou Medical University, Xuzhou, Jiangsu, China; ^5^ Department of Gastroenterology, Kunshan Third People’s Hospital, Suzhou, Jiangsu, China

**Keywords:** gastric cancer, chronic atrophic gastritis, machine learning, metabolism, single cell sequencing

## Abstract

**Background:**

Gastric cancer continues to be a significant global healthcare challenge, and its burden remains substantial. The development of gastric cancer (GC) is closely linked to chronic atrophic gastritis (CAG), yet there is a scarcity of research exploring the underlying mechanisms of CAG-induced carcinogenesis.

**Methods:**

In this study, we conducted a comprehensive investigation into the oncogenes involved in CAG using both bulk transcriptome and single-cell transcriptome data. Our approach employed hdWGCNA to identify pathogenic genes specific to CAG, with non-atrophic gastritis (NAG) serving as the control group. Additionally, we compared CAG with GC, using normal gastric tissue as the control group in the single-cell transcriptome analysis. By intersecting the identified pathogenic genes, we pinpointed key network molecules through protein interaction network analysis. To further refine the gene selection, we applied LASSO, SVM-RFE, and RF techniques, which resulted in a set of cancer-related genes (CRGs) associated with CAG. To identify CRGs potentially linked to gastric cancer progression, we performed a univariate COX regression analysis on the gene set. Subsequently, we explored the relationship between CRGs and immune infiltration, drug sensitivity, and clinical characteristics in gastric cancer patients. We employed GSVA to investigate how CRGs regulated signaling pathways in gastric cancer cells, while an analysis of cell communication shed light on the impact of CRGs on signal transmission within the gastric cancer tumor microenvironment. Lastly, we analyzed changes in metabolic pathways throughout the progression of gastric cancer.

**Results:**

Using hdWGCNA, we have identified a total of 143 pathogenic genes that were shared by CAG and GC. To further investigate the underlying mechanisms, we conducted protein interaction network analysis and employed machine learning screening techniques. As a result, we have identified 15 oncogenes that are specifically associated with chronic atrophic gastritis. By performing ROC reanalysis and prognostic analysis, we have determined that GADD45B is the most significant gene involved in the carcinogenesis of CAG. Immunohistochemical staining and differential analysis have revealed that GADD45B expression was low in GC tissues while high in normal gastric tissues. Moreover, based on prognostic analysis, high expression of GADD45B has been correlated with poor prognosis in GC patients. Additionally, an analysis of immune infiltration has shown a relationship between GADD45B and the infiltration of various immune cells. By correlating GADD45B with clinical characteristics, we have found that it primarily affects the depth of invasion in GC. Through cell communication analysis, we have discovered that the CD99 signaling pathway network and the CDH signaling pathway network are the main communication pathways that significantly alter the microenvironment of gastric tissue during the development of chronic atrophic gastritis. Specifically, GADD45B-low GC cells were predominantly involved in the network communication of the CDH signaling pathway, while GADD45B-high GC cells played a crucial role in both signaling pathways. Furthermore, we have identified several metabolic pathways, including D-Glutamine and D-glutamate metabolism and N-Glycan biosynthesis, among others, that played important roles in the occurrence and progression of GC, in addition to the six other metabolic pathways. In summary, our study highlighted the discovery of 143 pathogenic genes shared by CAG and GC, with a specific focus on 15 oncogenes associated with CAG. We have identified GADD45B as the most important gene in the carcinogenesis of CAG, which exhibited differential expression in GC tissues compared to normal gastric tissues. Moreover, GADD45B expression was correlated with patient prognosis and is associated with immune cell infiltration. Our findings also emphasized the impact of the CD99 and CDH signaling pathway networks on the microenvironment of gastric tissue during the development of CAG. Additionally, we have identified key metabolic pathways involved in GC progression.

**Conclusion:**

GADD45B, an oncogene implicated in chronic atrophic gastritis, played a critical role in GC development. Decreased expression of GADD45B was associated with the onset of GC. Moreover, GADD45B expression levels were closely tied to poor prognosis in GC patients, influencing the infiltration patterns of various cells within the tumor microenvironment, as well as impacting the metabolic pathways involved in GC progression.

## 1 Introduction

Gastric cancer is a highly prevalent malignant tumor of the digestive system, contributing significantly to cancer-related mortality worldwide (1). It exhibits epidemiological and histological variations across different nations, ranking as the fifth most commonly diagnosed cancer and the fourth leading cause of cancer-related deaths globally in 2020 (2). Lauren’s classification distinguishes GC into two major subtypes: intestinal GC and diffuse GC (3). Epidemiological evidence strongly associates chronic atrophic gastritis with the development of intestinal-type stomach cancer. CAG patients have an estimated annual risk of GC of 0.1 percent (4, 5). CAG, an inflammatory disease, is characterized by the loss of gastric glandular tissue in the stomach mucosa. This results in the replacement of the glandular structure with connective tissue (non-metaplastic atrophy) or an unidentifiable glandular structure (metaplastic atrophy) (6). CAG can be classified into two types based on its etiology: Helicobacter pylori-positive chronic atrophic gastritis and chronic autoimmune atrophic gastritis (CAAG). The majority of CAG-induced gastric cancer cases are associated with Helicobacter pylori infection (7, 8). The prevailing consensus is that the Correa cascade hypothesis provides a rough framework for understanding the cancerous progression from Helicobacter pylori-positive chronic inflammation of the gastric mucosa to CAG, intestinal metaplasia, dysplasia, and ultimately GC. Factors such as the highly acidic and irritating environment, direct damage to the stomach epithelium caused by Helicobacter pylori, and the conversion of nitrate in food into nitrite or n-nitroso compounds by Helicobacter pylori contribute significantly to the development of gastric cancer (9). Consequently, the progression from chronic inflammation to CAG, intestinal metaplasia, dysplasia, and ultimately GC is a complex and multistep process (10).

The immune microenvironment acts as a barrier comprising tumor cells, immune cells, fibroblasts, and other cells, along with their secreted immune factors (11–14). This complex network plays a dual role by suppressing tumor immunity and promoting tumor growth and dissemination (15–17). During tumor formation, tumor cells can dynamically modify their microenvironment through the secretion of various cytokines, chemokines, and other molecules. This reprogramming of neighboring cells enables them to actively contribute to tumor survival and growth (18). Chronic atrophic gastritis, whether caused by Helicobacter pylori infection or an autoimmune disorder, is characterized by the infiltration of immune cells in the gastric epithelium and deeper tissues. The interplay between these infiltrating immune cells, their secretion of inflammatory cytokines, and Helicobacter pylori virulence factors leads to persistent damage and repair reactions within the gastric epithelium, ultimately promoting the development of gastric cancer (19, 20). Inflammatory cells such as eosinophils and mast cells have been increasingly implicated in the carcinogenesis of chronic atrophic gastritis (21). Understanding the mechanisms underlying the immune microenvironment’s role in the initiation and progression of gastric cancer is of utmost importance, given its significant impact on tumor development. Therefore, investigating the intricate mechanisms governing gastric cancer has become a primary focus of research.

Machine learning offers significant research advantages in the identification of disease biomarkers by efficiently analyzing large and complex datasets, enabling the discovery of intricate patterns and relationships that may be overlooked by traditional methods (22–26). Besides, the adaptability of machine learning algorithms allows continuous learning from new data, leading to ongoing refinement and improvement of biomarker identification processes, ultimately enhancing diagnostic accuracy and precision (27–31). By integrating diverse data sources and leveraging advanced algorithms, machine learning facilitates the identification of disease-specific biomarkers associated with individual patient characteristics, enabling personalized medicine approaches and improved treatment selection for better patient outcomes (32–37). Previous studies on chronic atrophic gastritis and gastric cancer have been limited by research constraints, often relying on statistical analysis of epidemiological data and protein-level immunohistochemistry analysis. However, advancements in high-throughput sequencing technology, particularly single-cell sequencing, have provided unprecedented opportunities to unravel the intricate mechanisms underlying the formation and progression of gastric cancer (38). Unfortunately, the scarcity of chronic atrophic gastritis sequencing data has made the association between chronic atrophic gastritis and gastric cancer relatively rare. In this study, our objective is to leverage cutting-edge bioinformatics techniques to analyze single-cell transcriptome data of gastric cancer. By doing so, we aim to identify key genes that play crucial roles in the initiation, advancement, and regulation of the immune microenvironment in gastric cancer. Through this comprehensive analysis, we aim to shed new light on the role of chronic atrophic gastritis in the progression of gastric cancer, thus expanding our understanding of this complex disease.

## 2 Methods

### 2.1 Download and preliminary collation of the data used in this study

The single-cell sequencing data for CAG was obtained from the GEO database (registration number GSE134520). This dataset consisted of 13 experiments involving tissue sequencing. The submitter of the data utilized Cell Ranger software (version 2.2.0) for initial preprocessing, which involved removing low-quality cells. We specifically extracted sequencing information from three tissues with CAG and three tissues without atrophic gastritis for further analysis. Regarding stomach cancer, single-cell sequencing data was obtained from the GEO database with the registration number GSE163558. The original dataset included three cases of primary stomach cancer, one case of normal tissue, and six cases of gastric metastatic carcinoma. For initial data processing, Cell Ranger software (version 3.0.2) was used. We selected three cases of primary GC tissues and one case of normal tissues for subsequent data analysis. To interpret the single-cell transcriptome data, we employed the Seurat software (version 4.1.1). During the data reading process, a preliminary quality check was conducted using the following criteria: each gene had to be detectable in at least 50 cells, and each cell had to express at least three genes.

In this study, the TCGA (The Cancer Genome Atlas) database was utilized as the training set for obtaining common transcriptome data of stomach cancer. The data filtering process involved applying the following criteria: 1. The original location was “stomach”. 2. The platform was “TCGA-STAD”, and the disease type was “colonies and adenocarcinomas”. 3. The data category was “transcriptome profiling”. 4. The data type was “Gene Expression Quantification”. 5. There were no specific screening requirements for other options like gender, race, age, etc. To integrate, annotate genes, and eliminate redundancy in expression values, the original data were extracted. Samples with gene expression values of “0” were excluded after calculating the average expression value for each gene. A standard procedure, including transcript normalization and log2 conversion, was applied using the toil package to transform the RNA sequencing data into the format of per thousand bases per million fragments. This normalized data was then used for subsequent analysis. Consequently, the gene expression matrix of the TCGA cohort was obtained. Clinical data pertaining to matched patients, including their TCGA numbers, survival times (in days), and survival status (survival or death), were extracted. Patients under the age of 18 or with a survival time of less than 30 days were excluded from the study.

### 2.2 Further treatment of single cell matrix of chronic atrophic gastritis and gastric cancer

In the analysis of single-cell transcriptome data, Seurat was typically employed (11, 33). Initially, additional quality control steps were conducted to ensure the exclusion of low-quality data that could arise from cell separation-induced injury or library preparation failure. The following criteria were used for quality control: (1) The ratio of mitochondrial gene expression to total gene expression in each cell must be below 35%, and the top 2% of cells with the highest mitochondrial gene expression must be destroyed. (2) Exclusion of cells with gene expression levels of less than 500 or greater than 6000 (3) The UMI count value of the sequencing of each cell must be greater than 1000. (4) Calculated the proportion of rRNA expression in the complete gene and eliminated cells in the highest and lowest 1 percent. To mitigate batch effects during sample sequencing and ensure that downstream analysis is not influenced, we employed the Harmony approach from the harmony package (version 0.1.0) to integrate and debatch multiple samples. The data was then normalized using the NormalizeData function to account for differences in cell sequencing depths. Feature selection was performed using the FindVariableFeatures program, which identified 2000 highly variable genes for downstream analysis (34, 39). Linear dimension reduction was carried out using the RunPCA function, aiming to retain the characteristics of the original data while reducing the data dimensions. The ScaleData function was utilized to transform the gene expression values into z-scores following a Gaussian distribution. After the initial linear dimension reduction, the appropriate dimensions were selected for the final nonlinear dimension reduction using the UMAP (Uniform Manifold Approximation and Projection) method (40, 41). The data was projected onto two dimensions to achieve visualization. The FindNeighbors function was employed to construct a K-nearest neighbors (KNN) network based on the Euclidean distance in the PCA space. Edge weights between units were refined using their shared overlap (Jaccard similarity) in the local neighborhood, completing the final cell clustering. To enhance identification of common modular functionality, cell clusters were aggregated using the FindClusters function with a resolution of 0.5.

To ensure reliable findings, cell annotation involved a combination of automatic and manual annotation approaches. The automatic annotation was primarily performed using the singleR package (version 1.8.1), which predicted the potential cell types of individual cells based on a reference transcriptome dataset consisting of pure cell types. Manual annotation was based on the results of differential analysis. For the differential analysis, the FindAllMarkers function was utilized to identify genes that were differentially expressed between each subgroup and all other subgroups, using a filter criterion of a standard *P*-value < 0.05. The annotation results obtained from the singleR package were compared and complemented with additional information from sources such as CellMarker (42), the BMC Genome Biology online database, and comprehensive literature searches. This comprehensive approach led to the final annotation results for each cell cluster. To analyze the copy number distribution at the single-cell level, the copykat algorithm, which combined Bayesian techniques with hierarchical clustering, was employed. This algorithm enabled the identification of subclone structures and differentiation between benign and malignant cells based on the copy number distribution of individual cells. The variance of each cell population was calculated using a gaussian mixture model (GMM). High-confidence diploid cells were defined as the cell population with the minimum estimated variance, applying stringent categorization criteria. Hierarchical clustering of single-cell copy number data was performed to identify the largest gap between diploid normal cells and aneuploid tumor cells.

### 2.3 Identification of common pathogenic genes in gastric epithelial cells during CAG and GC based on single-cell transcriptome weighted gene co-expression network and inter-group difference analysis

We utilized the hdWGCNA package (version 0.2.2) developed by Sam Morabito et al. to perform weighted gene co-expression network analysis (hdWGCNA) on single-cell data (43, 44). The hdWGCNA method was highly modular and enabled the identification of robust gene modules and the construction of co-expression networks across multiple cell scales and hierarchical structures, specifically designed for single-cell sequencing data. To apply hdWGCNA, we first established a Seurat object using the SetupForWGCNA function (fraction = 0.05). Next, using the KNN technique, we aggregated the average or sum expression of these cell groups, resulting in a sparse metacell gene expression matrix. We defined the cell type consisting of malignant cells using the SetDatExpr function to generate the expression matrix. The TestSoftPowers function (networkType = ‘signed’) was then employed to perform parameter scans across various soft power thresholds (range from 1 to 30). By examining the network architecture at different power values, we determined the appropriate soft power threshold for constructing the co-expression network, ensuring a robust gene-gene adjacency matrix and eliminating weak links. In this study, a minimum soft power threshold greater than or equal to 0.9 was selected based on the fit to the scale-free topology model. The ConstructNetwork function (setDatExpr = FALSE) was used to create a co-expression network below the optimal soft threshold. To identify module feature genes, we applied the ModuleEigengenes function, which involved performing principal component analysis (PCA) on a subset of the gene expression matrix representing each module. This allowed us to obtain the module feature genes (ME) present in different modules. Additionally, the central gene feature score for each module was computed using the Seurat or UCell algorithms through the ModuleExprScore function (n_genes = 25). The ModuleCorrelogram function facilitated the visualization of the associations between modules based on their hME, ME, or hub gene scores. The importance of modules was determined using the GetModuleTraitCorrelation function, which conducted correlation analysis among modules and assessed their significance based on correlation coefficients and *P*-values. Heatmap visualization of module traits was achieved using the PlotModuleTraitCorrelation function. To investigate the inter-group differences of gastric epithelial cells, we conducted a differential gene expression analysis using the FindAllMarkers function. By examining the stomach epithelial cells in the single-cell matrix according to cell grouping data, we employed a screening criterion of *P* < 0.05 without setting a specific log fold change (logFC) threshold. This approach aimed to capture differentially expressed genes to the maximum extent possible while minimizing the generation of false negative findings.

### 2.4 Protein interaction network analysis identified key roles in the pathogenic gene network

We identified a set of oncogenes associated with CAG by intersecting four gene sets. To explore the protein-protein interactions (PPI) among these oncogenes, we utilized the STRING online database (accessed on March 2, 2022) and generated a protein interaction network. A confidence threshold of 0.4 was set as the default criterion for selecting reliable interactions. Subsequently, we imported the PPI results into Cytoscape 3.9.1, a network visualization and analysis software (28). To identify the most significant sub-networks within the PPI network, we applied the MCODE plug-in, which utilized the K-means clustering method. This analysis helped us identify key sub-networks containing vital genes within the context of CAG. By integrating the information from these key sub-networks and their constituent genes, we defined the potential network of oncogenes associated with CAG. This network provided insights into the molecular interactions and functional relationships among the identified oncogenes in the context of CAG.

### 2.5 The TCGA cohort’s most important genes were re-screened using three different machine learning approaches

Following the single-cell investigation, we identified a gene set associated with CAG and its carcinogenic potential. To prioritize the most important genes within this gene set, we employed three machine learning techniques. Firstly, we utilized the glmnet package (version 4.1.4) to perform the least absolute shrinkage and selection operator (LASSO) analysis (45, 46). This technique involved fitting a generalized linear model while performing variable selection and regularization. We determined the optimal lambda value, referred to as “lambda.min”, for the LASSO model (47, 48). Next, we employed the random forest algorithm using the randomForest program (version 4.7.1.1) (49, 50). This involved computing the average contribution value of each feature on multiple decision trees within the random forest. By comparing the contributions of various features, we ranked the top 30 genes. Lastly, we utilized the e1071 software (version 1.7.11) to implement support vector machine recursive feature elimination (SVM-RFE). SVM-RFE was a backward selection algorithm based on the support vector machine’s maximum margin principle. It allowed us to extract features and rank their significance in distinguishing tumor and non-tumor groups. The top 30 genes obtained from SVM-RFE analysis were included in the subsequent analysis (51). The genes selected through these three machine learning techniques were successfully validated using the TCGA cohort. These genes represented the most relevant and significant features associated with CAG in the context of carcinogenesis.

### 2.6 Univariate COX regression, KM survival curve, receiver operating characteristic curve and immunohistochemistry were used to find molecules related to gastric cancer progression

To identify genes that played a critical role in the carcinogenesis of CAG and the progression of gastric cancer, we employed several validation approaches. First, we utilized the receiver operating characteristic curve (ROC) analysis and immunohistochemistry to confirm the carcinogenic genes identified through machine learning. For the survival analysis, we used the “survival” and “survminer” packages (25, 52), setting the significance level at a *P* value of 0.05 (All *P*-value corrections in this study were performed using the default method, namely the Benjamini-Hochberg (BH) method). The TCGA cohort was divided into high- and low-expression groups based on the median value, and Kaplan-Meier (KM) analysis (24), employing the product-limit technique, was performed. The area under the ROC curve (AUC) was used to assess the diagnostic value of GC, with an AUC greater than 0.7 indicating higher diagnostic accuracy. The gene that passed both the survival analysis and ROC analysis was considered as the final gene associated with CAG and GC, referred to as CRG. To investigate the protein-level changes of CRG, we conducted an immunohistochemistry analysis using the human protein atlas. This analysis allowed us to track and visualize the expression of CRG protein in human tissue samples, providing further insights into its potential role in the development and progression of CAG and GC.

### 2.7 The effect of CRG expression on the immune microenvironment of gastric cancer patients

To investigate the impact of CRG on immune cell infiltration in the tumor microenvironment of gastric cancer, we employed the CIBERSORT deconvolution method. This computational tool utilizes linear support vector regression and a reference dataset of known immune cell subtypes to quantify immune infiltration based on the expression matrix. Using the concept of differential analysis, we compared immune cell infiltration between two patient groups. We examined the differences in immune cell composition and function within the immunological milieu of these two groups based on known functional annotations. Furthermore, we assessed the expression frequency of common immunological checkpoints in patients with different patterns of immune cell infiltration. To predict the responsiveness of patients with various immune cell infiltration patterns to immunotherapy, we utilized the online database called Tumor Immune Dysfunction and Exclusion (TIDE). This database provides valuable insights and predictions regarding the potential efficacy of immunotherapy in different patient populations. By integrating these analyses, we aimed to gain a better understanding of the relationship between CRG, immune cell infiltration, and the potential for immunotherapy response in gastric cancer patients.

### 2.8 The clinical importance of CRG expression for the diagnosis and treatment of patients with gastric cancer

To assess the clinical significance of CRG in gastric cancer patients, we obtained clinical data from the TCGA cohort and performed data filtering and merging with transcriptome data. This resulted in a dataset of 371 gastric cancer patients with integrated clinical and gene expression information. Subsequently, we conducted statistical analysis to reveal whether the expression levels of CRGs were significantly correlated with clinical parameters such as age, gender, differentiation degree, TNM stage, T, N, and M grouping of cancer patients (*P*<0.05).

There are a variety of treatments for tumors, including surgical resection, radiotherapy, chemotherapy, immunotherapy, and MRI-guided focused ultrasound (MRgFUS) (53). To investigate the sensitivity of CRG in GC patients to common chemotherapy regimens, we employed the pRRophetic algorithm. This algorithm utilizes a ridge regression model that combines the GDSC cell line expression profile and the TCGA gene expression profile. It predicted the half-inhibitory concentration of the drug (IC50), which represented the drug concentration at which 50% of cells undergo apoptosis, based on the drug sensitivity analysis developed by Paul Geeleher et al. in 2014 (54). To illustrate the results of the drug sensitivity analysis, we used box plots to visualize the dispersion of IC50 values across multiple samples. Additionally, we examined the correlation between the model score and IC50 to assess the relationship between CRG expression and drug sensitivity (55). By conducting these analyses, we aimed to explore the clinical implications of CRG in gastric cancer patients, including its associations with patient characteristics and its potential role in predicting the response to common chemotherapy regimens.

### 2.9 Trajectory analysis of CRG high/low expression gastric cancer cells and their specific transcription factors

We proceeded to investigate the influence of CRG expression on individual GC cells. To achieve this, we isolated GC cells from the single-cell matrix of GC samples and performed a reclassification process consistent with the initial classification. Refinement of the classification was based on the expression of cell-specific markers, cell cycle genes, and CRG. This allowed us to distinguish between gastric cancer stem cells, cancer cells with high CRG expression, and cancer cells with low CRG expression.

To infer the differentiation trajectory of GC cells and understand the evolution of cell subtypes during development, we conducted pseudo-time series analysis using the monocle package (version 2.22.0). This analysis involved assessing changes in gene expression across various GC cell subsets over time. We employed the detectGenes function to eliminate low-quality cells (expression threshold = 0.1) and computed size factors and dispersions. After selecting the top 200 differentially expressed gene clusters, we used the reduceDimension function’s DDRTree method to reduce the dimensionality of the data (56). We then calculated the developmental time, inferred the trajectory, sorted the cells based on the pseudo-time, and visualized the results using a tree diagram. The branching pattern of GC stem cells helped determine the starting point of the differentiation trajectory.

For predicting the activity of transcription factors (TFs), we utilized the “DoRothEA” package, which incorporated a gene regulatory network consisting of symbolic TF-target gene interactions. Initially, we employed the run_viper function to calculate the viper score for each regulator gene in the DoRothEA network. Next, we incorporated the TF activity matrix into the cell clustering and dimension reduction process, following the same methods as before. We further computed the TF activity fraction for each cell subset. Finally, we visualized the 90 TFs with the most significant changes between CRG high-expression and low-expression gastric cancer cells using a heatmap, based on their TF scores.

By employing these analytical approaches, we aimed to gain insights into the differentiation trajectory and cellular dynamics of gastric cancer cells, as well as predict the activity of TFs associated with CRG expression in gastric cancer cells.

### 2.10 Identified the important signaling pathways of CRG mediated chronic atrophic gastritis carcinogenesis

To identify the CRG involved in the activation of signaling pathways in gastric cancer cells, we performed Gene Set Variation Analysis (GSVA) on gastric cancer tissues obtained from the TCGA cohort. Additionally, we extracted epithelial cells from the single-cell matrix of CAG and performed GSVA analysis on two groups of epithelial cells based on their grouping information (non-atrophic gastritis vs. chronic atrophic gastritis). Similarly, using the single-cell matrix of GC, we performed GSVA analysis on epithelial cells according to their grouping information (normal gastric tissue vs. GC tissue). By comparing the results of the three analyses, we identified signaling pathways that exhibited consistent trends across all three datasets. These pathways, showing consistent activation or inhibition in association with CRG-mediated chronic atrophic gastritis carcinogenesis, were considered essential signaling pathways implicated in the disease.

### 2.11 The effect of gastric cancer cells with high/low expression of CRG on intercellular communication in tumor microenvironment

To investigate the impact of high or low CRG expression in GC cells on cell-to-cell communication within the tumor microenvironment, we conducted a cell signal communication analysis. Cell-to-cell communication referred to the exchange of information between cells through chemical signal molecules, which played a crucial role in regulating various biological processes such as development, differentiation, and inflammation (28–31, 57). In this study, we utilized the “CellChat” software (version 1.1.3) to infer and analyze the intercellular interaction network. First, we identified the overexpressed ligands or receptors within specific cell groups. Then, we determined the interactions between the overexpressed ligands and receptors. The communication probability of all ligand-receptor interactions associated with each signaling route was inferred, and the aggregate communication network between cells was established by considering the linkages or the aggregate communication probability. By projecting the gene expression data into a PPI network, we constructed a comprehensive network of cell communication at the ligand-receptor and signaling pathway levels. This analysis allowed us to gain insights into the complex interplay and communication among different cell types within the gastric cancer microenvironment.

### 2.12 Identifying metabolic changes in chronic atrophic gastritis and gastric cancer using scMetabolism

To investigate the metabolic characteristics of gastric cancer tissue at the single-cell level, we conducted metabolic analysis using the scMetabolism package (58). This package provided a comprehensive metabolic module that allowed us to annotate and quantify metabolites within each individual cell. By combining information from public metabolite databases and relevant literature, we determined the specific types and quantities of metabolites present in each cell. Next, we mapped the annotated metabolites to well-established metabolic pathway databases such as kyoto encyclopedia of genes and genomes (KEGG). Through enrichment analysis methods, we evaluated the enrichment of metabolic pathways in individual cells or across multiple cells. This analysis enabled us to explore the biological functions associated with different metabolic pathways and gain insights into the composition of metabolic networks within GC tissue. By employing the scMetabolism package and leveraging existing knowledge on metabolites and metabolic pathways, we were able to shed light on the metabolic processes and their potential implications in GC at the single-cell level.

### 2.13 Cell culture

The normal gastric mucosal cell line GES-1 and gastric cancer cell lines AGS, HGC-27, MKN28, and MKN45 were obtained from the Cell Resource Center of Peking Union Medical College. These cell lines were cultured in DMEM medium supplemented with 10% FBS and 1% penicillin sodium and streptomycin. The cells were maintained in a constant temperature incubator set at 37°C with a 5% CO2 atmosphere.

### 2.14 Real-time quantitative PCR and immunofluorescence staining

For reverse transcription, the HiScript III first-strand cDNA synthesis kit (Nanjing, China) was employed following the manufacturer’s instructions. Real-time PCR was conducted on the ABI StepOne PlusTM system using SYBR® Green for Master Mix (Vazyme, Nanjing, China). Relative mRNA expression was calculated using the 2-CT method, with triplicate measurements performed. The primer sequences can be found in **Supplementary Table 1**.

The study protocol was approved by the Ethics Committee of the First Affiliated Hospital of Soochow University and adhered to the guidelines outlined in the Helsinki Declaration. Paraffin sections of gastric cancer and adjacent normal tissues were obtained from the First Affiliated Hospital of Soochow University. The sections underwent dewaxing, hydration, antigen repair, and blocking procedures. Immunofluorescence staining of GADD45B was performed in Seville, Wuhan, China. The number of GADD45-B-positive cells in cancer and adjacent tissues was determined using fluorescence microscopy.

### 2.15 CCK8 experiment

The cancer cell lines AGS, HGC-27, MKN28, and MKN45 were maintained in sterile conditions until they reached the logarithmic growth phase, after which they were washed with sterile PBS. The cells were then seeded into 96-well plates at a specific density and incubated under constant temperature and humidity until they reached a stable state of attachment and growth. To assess the effects of the tested factors on cell proliferation and survival, CCK-8 reagent was added to the cell culture medium at a specific ratio (59). The cells were incubated for 4 hours under constant temperature and humidity. Following the incubation period, the absorbance values of the different treatment groups and the control group were measured using a multifunctional microplate reader. The relative proliferation rate of the different treatment groups was calculated to evaluate the impact of the tested factors on cell proliferation and survival.

### 2.16 Cell scratch test

The appropriate cell lines were cultured until they reached the logarithmic growth phase, and then a specific density of cells was seeded in 6-well or 12-well plates and incubated until they reached a stable state. A straight line was created on the cell monolayer using a cell scraper to generate a wound area. Subsequently, the cells were treated with the test compound or other factors at different concentrations, and both control and experimental groups were established. The cells were further incubated under constant temperature and humidity conditions for a specific period to allow for cell migration. To assess cell migration and invasion, the cells within the wound area were stained with various cell staining reagents, and the migration ability was observed and captured under a microscope. The evaluation of the test factors’ effect on cell migration involved calculating the area not occupied by cells in the wound area or measuring the distance of cell movement, among other indicators.

### 2.17 Online website

GEO: https://www.ncbi.nlm.nih.gov/geo/

CellMarker: http://xteam.xbio.top/CellMarker/

BMC Genome Biology: https://genomebiology.biomedcentral.com/

TCGA: https://www.cancer.gov/about-nci/organization/ccg/research/structural-genomics/tcga

STRING: https://string-db.org/

The human protein atlas: https://www.proteinatlas.org/

CIBERSORTx: https://cibersortx.stanford.edu/

TIDE: http://tide.dfci.harvard.edu/

## 3 Results

### 3.1 Single cell sequencing analysis revealed the unique immune microenvironment landscape of CAG and GC

**Figure 1:**
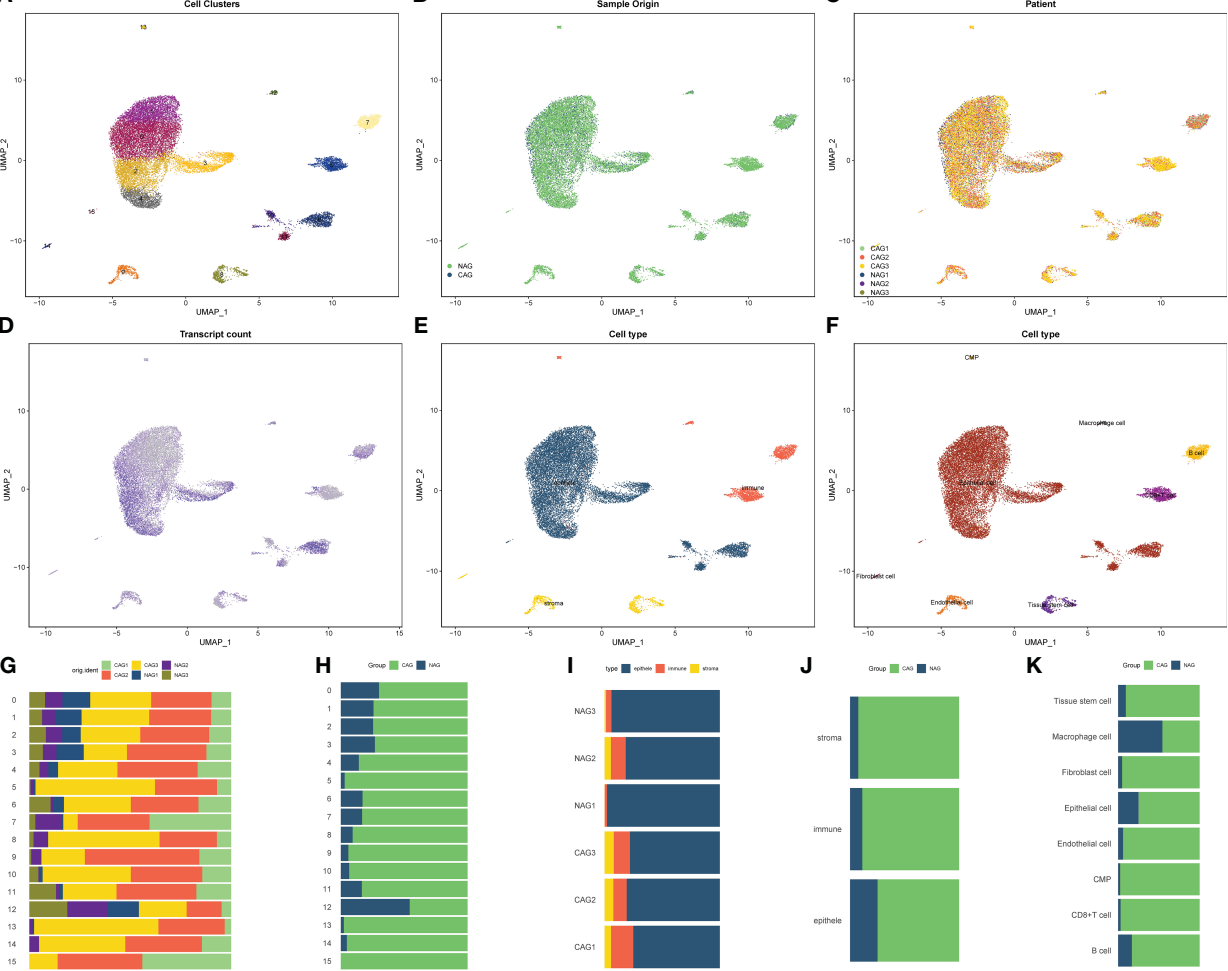
Microenvironment landscape of CAG and NAG. **(A)** The distribution of 16 cell subsets after UMAP dimension reduction. **(B)** Cell distribution in CAG and NAG. **(C)** Distribution of all cells in 6 tissue samples. **(D)** The gene expression of each cell-the darker the color, the higher the expression of the cell. **(E)** Preliminary classification of gastritis tissue. **(F)** Eight cell subtypes after annotation. **(G–K)** The proportion of different cell division methods from different tissue/sample sources. **(G)** The proportion of 16 cell subsets in 6 source tissues. **(H)** The proportion of 16 cell subsets in CAG and NAG tissues. **(I)** The proportion of three microenvironment components in six source tissue samples. **(J)** The proportion of three microenvironment components in CAG and NAG tissues. **(K)** The proportion of eight cell subsets in CAG and NAG tissues.

We initiated the processing and analysis of the sequencing data for CAG. The raw data consisted of 29,678 cells and 19,823 genes. After conducting quality control, we obtained a Seurat matrix comprising 20,151 cells and 19,823 genes (**Supplementary Figure 1**). Subsequently, we selected the first 30 principal components for further exploration through PCA. Utilizing UMAP dimensionality reduction, we identified 16 distinct cell subsets (**Figure 1A**). Among these subsets, there were 4,511 cells from non-atrophic gastritis tissues and 15,640 cells from chronic atrophic gastritis tissues (**Figures 1B, C**). Each cell subset exhibited varying levels of gene expression, with subgroups 2, 3, 4, and 6 demonstrating higher gene expression compared to subgroups 1, 5, 8, 10, and 13, while subgroups 2, 3, 4, and 6 exhibited lower gene expression (**Figure 1D**). To further classify the cell subsets, we utilized marker genes associated with tumor parenchyma and interstitial components (**Figure 1E**). By annotating each subgroup, we were able to distinguish the cell types within each subgroup, leading to the identification of eight distinct cell types (**Figure 1F**). For a comprehensive understanding of the subsets, cell proportions, cell variations, as well as tissue and sample distributions, we provided detailed figures in **Figures 1G–K**.

**Figure 2:**
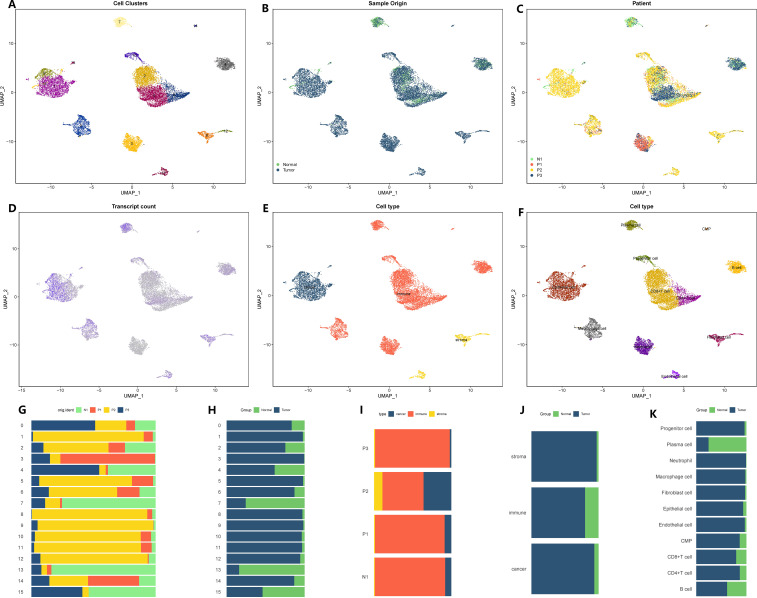
Microenvironment landscape of GC and non-tumor tissues. **(A)** The distribution of 16 cell subsets after UMAP dimension reduction. **(B)** Cell distribution in GC tissues and normal gastric tissues. **(C)** Distribution of all cells in 4 tissue samples. **(D)** The gene expression of each cell-the darker the color, the higher the expression of the cell. **(E)** Preliminary classification of gastritis tissue. **(F)** Eleven cell subtypes after annotation. **(G–K)** The proportion of different cell division methods from different tissue/sample sources. **(G)** The proportion of 16 cell subsets in 4 source tissues. **(H)** The proportion of 16 cell subsets in GC tissues and normal gastric tissues. **(I)** The proportion of three microenvironment components in 4 source tissue samples. **(J)** The proportion of three microenvironment components in GC tissues and normal gastric tissues. **(K)** The proportion of eight cell subsets in GC tissues and normal gastric tissues.

We proceeded to apply the same analysis to the sequencing data for stomach cancer. The initial GC matrix consisted of 18,223 cells and 24,159 genes. After conducting quality control, we retained 13,641 cells and 24,159 genes for further analysis. By utilizing a similar approach, we divided the total cell population into 16 distinct subgroups (**Figure 2A**). Among these subgroups, 11,425 cells originated from GC tissues, while 2,216 cells originated from healthy stomach tissues (**Figures 2B, C**). Subgroups 0, 2, 3, and 4 exhibited lower levels of gene expression, while subgroups 1, 8, 7, and 13 displayed higher levels of gene expression (**Figure 2D**). Further examination of the cellular composition revealed that the majority of cells within the stomach cancer microenvironment were immune cells, while interstitial cells were less abundant (**Figure 2E**). Through annotation, we identified eleven distinct cell types (**Figure 2F**). Additionally, utilizing the Copykat algorithm, we identified aneuploid cells that shared similarities with the cell subsets defined by the tumor marker gene “EPCAM,” all of which originated from epithelial cells (**Figure 2F**). For a comprehensive overview of subset proportions, cell variations, as well as tissue and sample distributions, please refer to **Figures 2G–K**. In this study, the identified cells and the corresponding cell markers were shown in **Supplementary Figure 2**.

### 3.2 The common pathogenic genes of CAG and GC were identified by hdWGCNA

**Figure 3:**
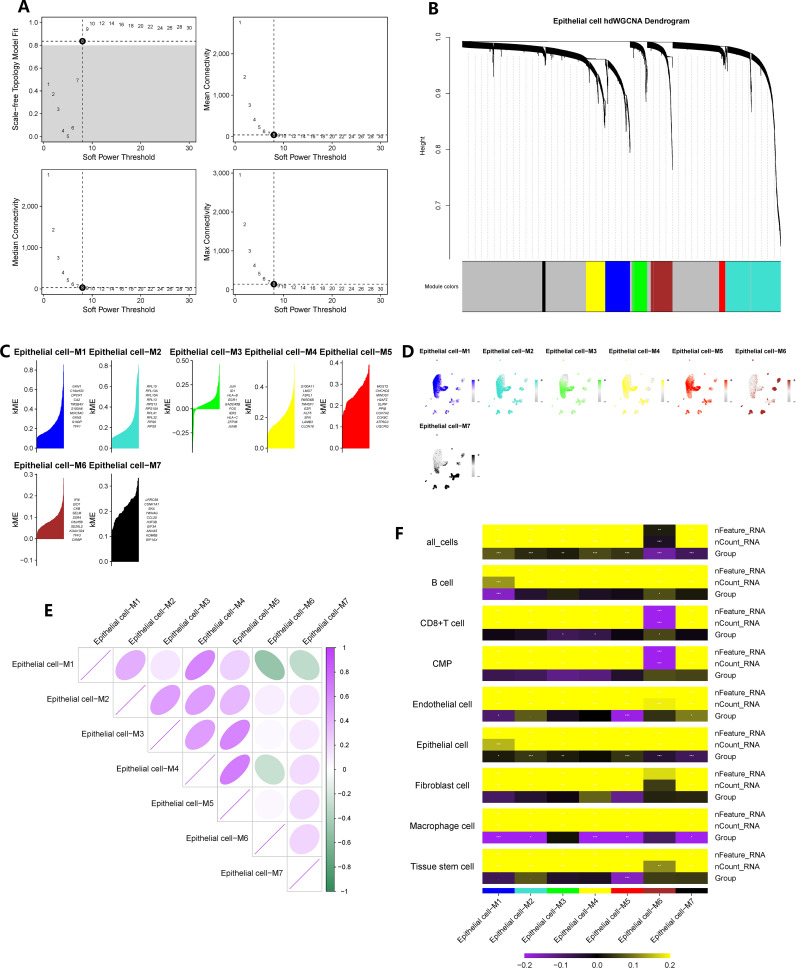
hdWGCNA has identified the key epithelial cell genes in the pathogenesis of chronic atrophic gastritis. **(A)** The selection of the optimal soft threshold. **(B)** Build a co-expression network based on the ideal “8” soft threshold and divide the genes into several modules to get a gene clustering tree. **(C)** Calculate the feature-based gene connectivity of each gene in the co-expression network analysis to determine the highly connected genes in each module. **(D)** Calculate gene scores for each module gene based on the UCell algorithm. **(E)** Correlation heatmap between modules. **(F)** The correlation heatmap between grouping information (chronic atrophic gastritis vs. non-atrophic gastritis) and epithelial cells. * represent P < 0.05, ** represent P < 0.01, and *** represent P < 0.001.

We constructed a co-expression network based on single-cell data using the optimal soft threshold of 8, as shown in **Figure 3A**. The module interconnectivity (kME) was evaluated based on the characteristic genes of each module (**Figure 3B**). As a result, we identified seven non-gray modules and their corresponding distinct genes (**Figure 3C**). The enrichment fraction of these distinctive genes in each cell subgroup was visualized in **Figure 3D**. The correlation between modules was depicted in **Figure 3E**. Notably, all seven modules, consisting of 2421 genes, showed strong associations with epithelial cells, as indicated by the heatmap in **Figure 3F**. Moreover, a comparison of epithelial cells between CAG and NAG groups revealed 519 differentially expressed genes.

**Figure 4:**
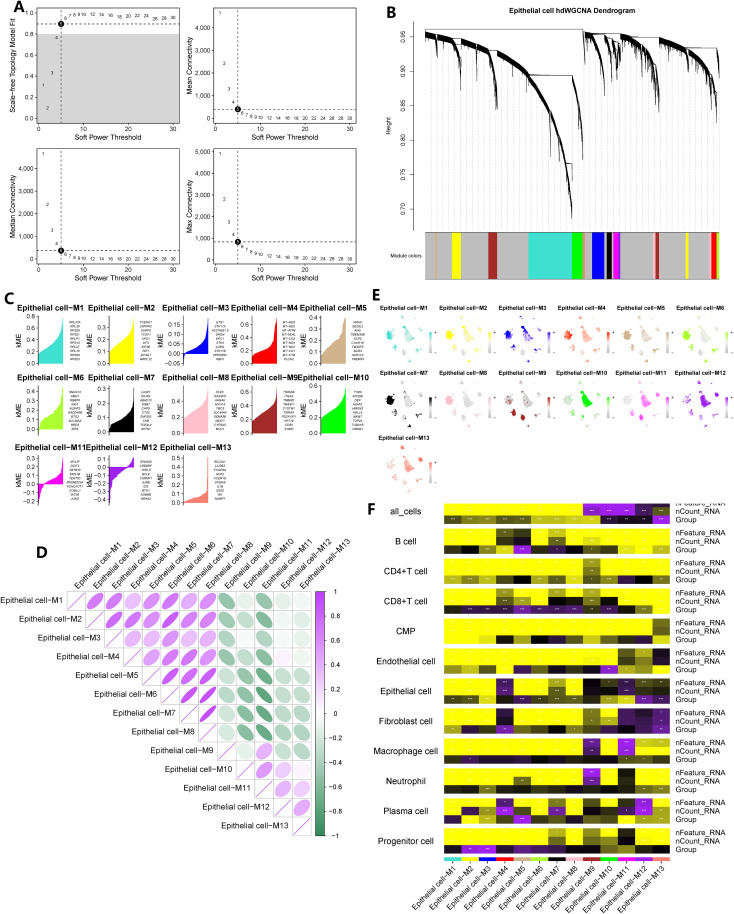
hdWGCNA has identified the key epithelial cell genes in the pathogenesis of gastric cancer. **(A)** The selection of the optimal soft threshold. **(B)** Build a co-expression network based on the ideal “5” soft threshold and divide the genes into several modules to get a gene clustering tree. **(C)** Calculate the feature-based gene connectivity of each gene in the co-expression network analysis to determine the highly connected genes in each module. **(D)** Calculate gene scores for each module gene based on the UCell algorithm. **(E)** Correlation heatmap between modules. **(F)** The correlation heatmap between grouping information (GC and normal tissues) and epithelial cells. * represent P < 0.05, ** represent P < 0.01, and *** represent P < 0.001.

In the case of GC, we utilized a soft threshold of 5 to construct a co-expression network (symbolic network), as depicted in **Figure 4A**. Thirteen modules were identified within the GC tissue co-expression network (**Figures 4B, C**). The enrichment scores and correlations of distinctive genes within these 13 modules were displayed in **Figures 4D, E**. However, module 9 did not exhibit statistically significant associations with epithelial cells based on the correlation heatmap. Hence, we focused on the remaining 12 modules, which encompassed a total of 3337 genes, for further investigation. Additionally, by performing differential analysis between normal and cancerous stomach tissues, we identified 2852 differentially expressed genes within the epithelial cell population.

### 3.3 Protein-protein interaction network analysis provided a key subnetwork of common pathogenic genes

**Figure 5:**
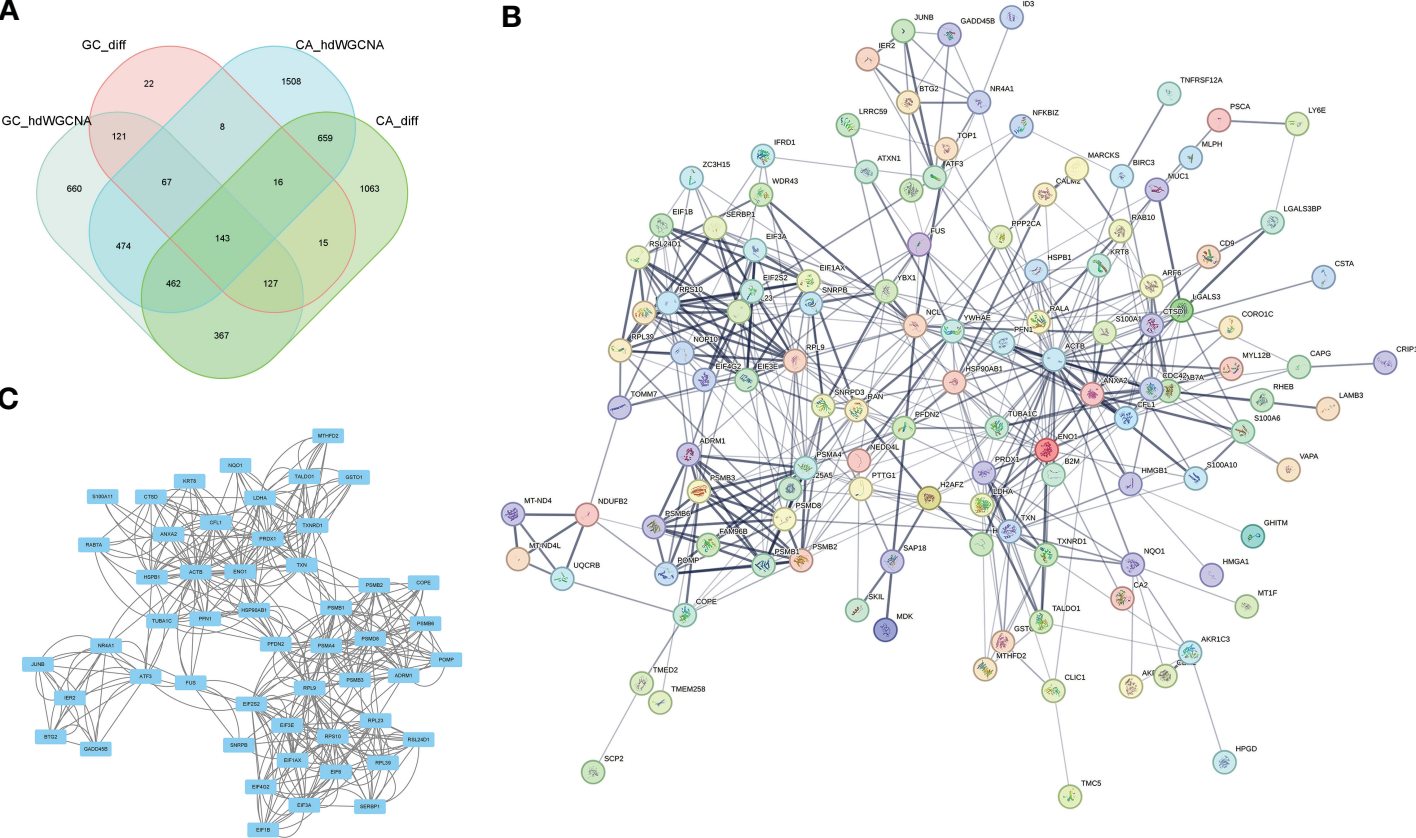
PPI analysis identified the core genes in the common pathogenic gene network of CAG and GC. **(A)** In two sets of single-cell transcriptome data, the intersection of important module genes and differentially expressed genes. **(B)** The protein interaction network constructed for the intersection genes on the String online website. **(C)** MCODE identified the key sub-networks in the intersection network.

Following the integration of the four datasets from the previous step, we identified a total of 143 potential oncogenes associated with CAG, as illustrated in **Figure 5A**. The PPI network of these 143 genes was visualized using the STRING online website (**Figure 5B**). By utilizing Cytoscape’s MCODE plug-in, we extracted the most significant subnetwork from the entire network, which consisted of 51 key molecules (**Figure 5C**).

### 3.4 Three machine learning methods identified 15 oncogenes of CAG

**Figure 6:**
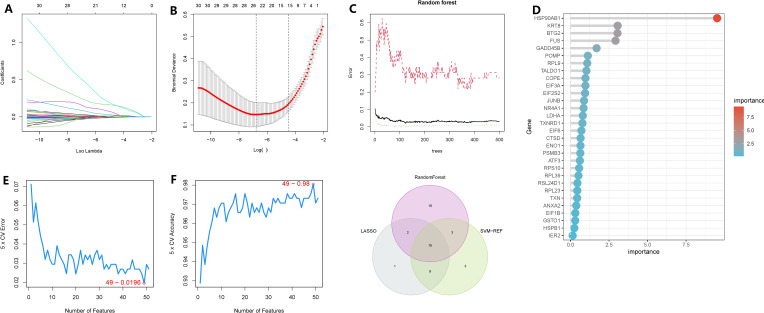
Identification of oncogenes in chronic atrophic gastric cancer based on three machine learning methods. **(A, B)** LASSO regression selected 28 variables based on the minimum lambda value. **(C, D)** random forest sorts the importance of all genes. **(E, F)** SVM-RFE evaluated all genes using a ten-fold cross-validation method and ranked the important gene rows according to the average ranking. **(G)** The Venn graphic illustrates the results shared by the three screening procedures.

To minimize the false-positive rate of our findings, we employed three machine learning methods to identify the key genes within the subnetwork. Firstly, using the LASSO regression model, we selected lambda.min and identified 26 genes as significant (**Figures 6A, B**). Subsequently, through random forest analysis, we ranked the genes based on their importance and extracted the top 30 genes (**Figures 6C, D**). Additionally, using SVM-RFE with 10-fold cross-validation, we obtained the top 30 genes based on their average ranking across the folds (**Figures 6E, F**). Ultimately, 15 genes were consistently identified as significant by all three machine learning algorithms: HSP90AB1, FUS, CTSD, KRT8, TALDO1, BTG2, TXNRD1, GADD45B, PSMB3, RPL9, NQO1, MTHFD2, CFL1, PRDX1, and PFDN2.

### 3.5 Prognostic analysis, ROC curve and human protein atlas identified GADD45B as an oncogene of chronic atrophic gastritis and related to the prognosis of patients with GC

**Figure 7:**
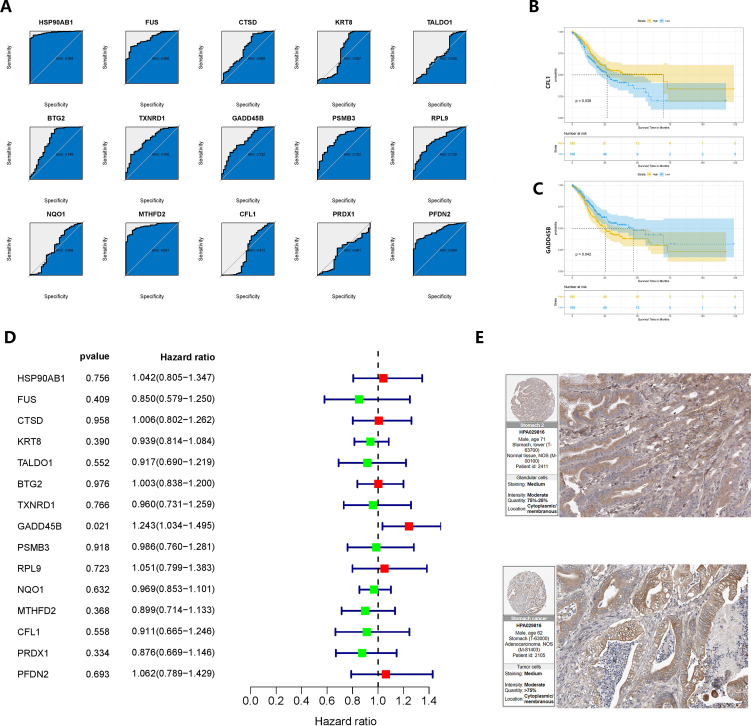
Verification and re-screening of oncogenes in chronic atrophic gastric cancer. **(A)** The diagnostic value of CRGs was determined using ROC analysis. **(B, C)** Survival analysis based on the product limit method and log-rank test Grouping is based on the median value of the expression. **(D)** Prognostic analysis based on univariate COX regression. **(E)** Immunohistochemical results of GADD45B on the human protein atlas.

The baseline data of pancreatic cancer patients in the TCGA cohort were presented in **Supplementary Table 2**. To identify oncogenes associated with the development of gastric cancer in CAG, we analyzed 15 genes. Among them, HSP90AB1, FUS, BTG2, TXNRD1, GADD45B, PSMB3, RPL9, MTHFD2, and PFDN2 showed area under the curve (AUC) values greater than 0.7 in ROC analysis, indicating their potential diagnostic value (**Figure 7A**). In the KM survival analysis, CFL1 and GADD45B were found to be correlated with patients’ prognosis (*P* = 0.038 and *P* = 0.042, respectively) (**Figures 7B, C**). Furthermore, GADD45B (*P* = 0.021) was identified as a significant risk factor for survival in GC patients through univariate COX regression (60), while the other genes did not show such an association with prognosis (**Figure 7D**). Based on these findings, we concluded that GADD45B was a prominent oncogene linked to CAG and was significantly associated with the prognosis of GC patients, suggesting its potential involvement in regulating GC progression. The expression of GADD45B in the human protein atlas revealed moderate staining intensity in normal tissues and low to moderate staining intensity in malignant tissues (**Figure 7E**).

### 3.6 Immune infiltration analysis showed the effect of GADD45 B on the immune microenvironment of GC patients and the prediction of the effect of immunotherapy

**Figure 8:**
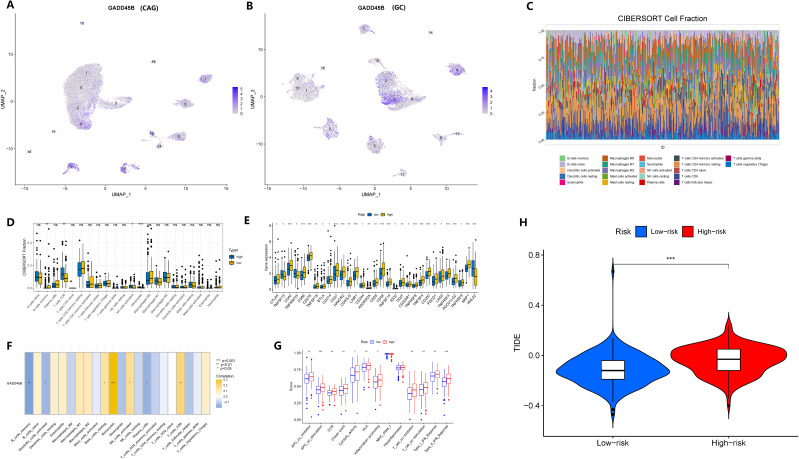
Effect of GADD45B on cell infiltration and function of GC immune microenvironment. **(A, B)** The expression of GADD45B in the microenvironment of CAG and GC. **(C, D)** Depending on the level of GADD45B expression, the degree of immune cell infiltration varied between the two groups. **(E)** Immune checkpoint expression levels differ between the two groups. **(F)** GADD45B and immune cell correlation. **(G)** Differences in immune function between the two groups. **(H)** Prediction of the efficacy of immunotherapy in two groups of gastric cancer patients. "ns" represent P > 0.05. * represent P < 0.05, ** represent P < 0.01, and *** represent P < 0.001.

Given that chronic atrophic gastritis was an inflammatory disease, we identified GADD45B as a crucial oncogene that might contribute to the development of GC. We hypothesized that GADD45B might play a significant role in regulating the tumor microenvironment in GC. Analyzing the single-cell matrix of CAG and GC, we observed high expression of GADD45B in various immune cells (**Figures 8A, B**). Based on the expression level of GADD45B, we conducted an immune infiltration study using data from the TCGA cohort of GC. The analysis revealed a correlation between GADD45B expression and the infiltration of B cells, CD8+ T cells, monocytes, and NK cells (**Figures 8C, D**). The upregulation of several known immune checkpoints was observed in the high-expression group of GADD45B, suggesting more severe immunosuppression in the tumor microenvironment of patients with high GADD45B expression (**Figure 8E**). The differential analysis and correlation analysis yielded consistent results, indicating a potential impact of GADD45B expression on the infiltration of plasma cells, mast cells, and dendritic cells (**Figure 8F**). Furthermore, GC tissues with high expression of GADD45B frequently exhibited immune checkpoint upregulation and significant suppression of anti-tumor immunity, as evidenced by statistically significant differences (**Figure 8G**). Considering this immunological dysfunction and substantial suppression of anti-tumor immunity, immunotherapy might hold promise as a significant treatment for patients with GC displaying high expression of GADD45B (**Figure 8H**).

### 3.7 Drug sensitivity analysis demonstrated the effect of GADD45B on drug therapy in patients with gastric cancer

**Figure 9:**
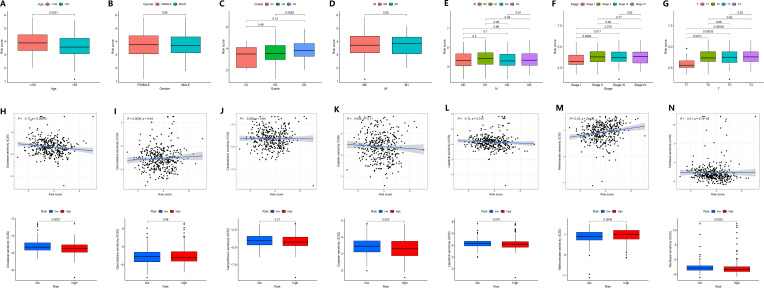
Correlation of clinical features and drug sensitivity analysis of GADD45B. **(A–G)** Correlation of clinical features of GADD45B. **(A)** Age. **(B)** Gender. **(C)** Grade. **(D)** Distant metastasis. **(E)** Lymph node metastasis. **(F)** TNM staging. **(G)** Primary tumor infiltration. **(H–N)** The effect of GADD45B on drug efficacy. **(H)** Docetaxel. **(I)** Gemcitabine. **(J)** Camptothecin. **(K)** Cisplatin. **(L)** Lapatinib. **(M)** Methotrexate. **(N)** Paclitaxel.

We investigated the correlation between GADD45B expression and clinical characteristics in GC patients. The data revealed that GADD45B expression was frequently higher in patients under 65 years old compared to those over 65 years old (**Figure 9A**). No significant difference in GADD45B expression was observed between males and females (**Figure 9B**). GADD45B had an impact on cancer differentiation, especially in patients with G2 and G3 stage tumors, where there were differences in the expression levels of GADD45B (**Figure 9C**). Furthermore, GADD45B expression was not associated with hematogenous or lymph node metastasis in GC (**Figures 9D, E**). Its main effect on TNM staging was seen in the depth of tumor invasion, contributing to the overall staging (**Figures 9F, G**).

Moreover, GADD45B expression was found to potentially influence the efficacy of chemotherapy in stomach cancer patients. Drug sensitivity analysis indicated an inverse relationship between the expression level of GADD45B and therapeutic sensitivity to docetaxel, lapatinib, and paclitaxel, suggesting that patients with low or high GADD45B expression might experience increased efficacy with these drugs. Additionally, a positive correlation was observed between the sensitivity to methotrexate treatment and GADD45B expression level, indicating potential increased efficacy in patients with high GADD45B expression. However, GADD45B expression level did not correlate with the therapeutic sensitivity of gemcitabine, camptothecin, and cisplatin (**Figures 9H–N**).

### 3.8 The reclassification of GC cell subsets revealed the effect of GADD45B on the differentiation trajectory and specific transcription factors of GC cells

**Figure 10:**
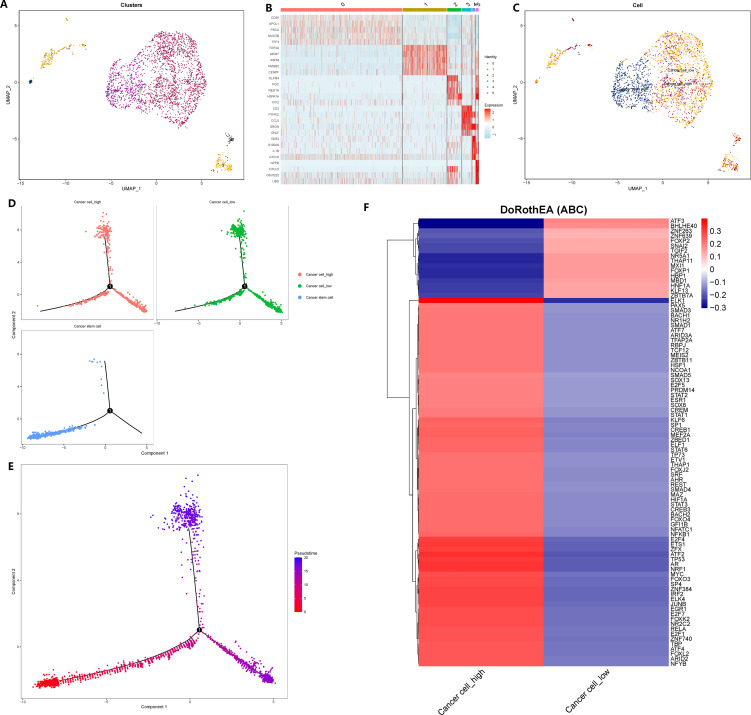
Reclassification of gastric cancer cells, cell trajectory analysis and transcription factor prediction. **(A)** The epithelial cells in the gastric cancer matrix were extracted and reclassified into 5 cell subsets. **(B)** The marker genes of each cell subgroup. **(C)** Fine annotation of tumor cells. **(D)** Differentiation trajectories of three cell types. **(E)** Differentiation trajectories of cell differentiation and maturation. **(F)** The most variable transcription factor between GADD45B-high GC cells and GADD45B-low GC cells.

To explore the impact of GADD45B on GC cells, we isolated and reclassified the epithelial cells from the single-cell matrix of GC tissue. After performing dimensionality reduction and clustering, we identified six distinct cell subsets (**Figure 10A**). Subgroup 1 exhibited elevated expression levels of cell cycle genes PCNA and MKI67, as well as the tumor stem cell marker CD44 (**Figure 10B**). Therefore, we classified subgroup 1 as cancer stem cells and subgroups 0, 2, 3, and 5 as common tumor cells (**Figure 10C**). Next, we stratified the tumor cells based on the expression level of GADD45B. Tumor cells with expression values above the median were categorized as GADD45B-high, while those with expression values below the median were classified as GADD45B-low. To investigate the impact of GADD45B on the differentiation trajectory of tumor cells, we performed pseudo-time series analysis on these three cell types. Analysis showed that the differentiation trajectories of GADD45B-high tumor cells and GADD45B-low tumor cells were essentially the same (**Figures 10D, E**), but further Beam tests indicated that GADD45B played an important role in the differentiation and development process of GC cells (P<0.05). Furthermore, we identified the most significant transcription factors associated with GADD45B-high tumor cells and GADD45B-low tumor cells. GADD45B-high tumor cells showed strong associations with transcription factors such as ELK1, PAX5, SMAD3, BACH1, and NR1H2. On the other hand, ATF3, BHLHE40, ZNF263, and FOXP2 transcription factors were strongly associated with GADD45B-low tumor cells (**Figure 10F**).

### 3.9 Enrichment analysis of single cell transcriptome and bulk transcriptome based on KEGG database identified significant differences in WNT signaling pathway in cancer cells with high/low expression of GADD45B

**Figure 11:**
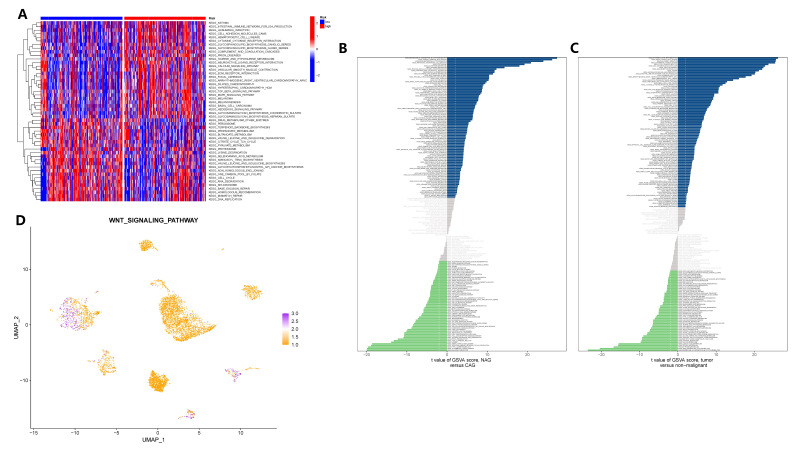
GSVA analysis identified carcinogenic signaling pathways in gastric epithelial cells. **(A)** GSVA analysis after grouping according to GADD45B expression level in the TCGA cohort. **(B)** The signaling pathway of significant changes in epithelial cells during the pathogenesis of CAG. **(C)** Signaling pathways in which epithelial cells undergo significant changes during the pathogenesis of GC. **(D)** The activation level of the WNT signaling pathway in each cell of the GC tumor microenvironment.

To elucidate the signaling pathway through which GADD45B controls gastric epithelial cells and contributes to the carcinogenic process of CAG, we conducted analyses using bulk transcriptome and single-cell transcriptome data. The GSVA was applied to both datasets. In the TCGA cohort, GSVA results revealed that the TGF-BETA signaling pathway, MAPK signaling pathway, JAK-STAT signaling pathway, and WNT signaling pathway exhibited distinct activities in GC tissues with different expression levels of GADD45B (**Figure 11A**). Analyzing the single-cell matrix of epithelial cells from CAG patients, GSVA showed high expression of the PPAR, MTOR, Neurotrophin, and WNT signaling pathways in gastric epithelial cells (**Figure 11B**). In the single-cell matrix of gastric cancer, GSVA demonstrated high activity in the P53, TOLL-LIKE RECEPTOR, and WNT signaling pathways (**Figure 11C**). Notably, the WNT signaling pathway emerged as crucial in the pathogenesis of CAG and GC, exhibiting a strong inverse correlation with GADD45B expression. Lastly, we assessed the activation level of the WNT signaling pathway in the tumor microenvironment, where GC cells prominently displayed its activation (**Figure 11D**). In conclusion, the WNT signaling pathway was identified as the pivotal pathway through which GADD45B regulated the carcinogenesis of CAG.

### 3.10 Cell communication analysis of CAG and GC single-cell transcriptomes revealed the effect of GADD45B on tumor cell signal transduction in the immunological microenvironment

**Figure 12:**
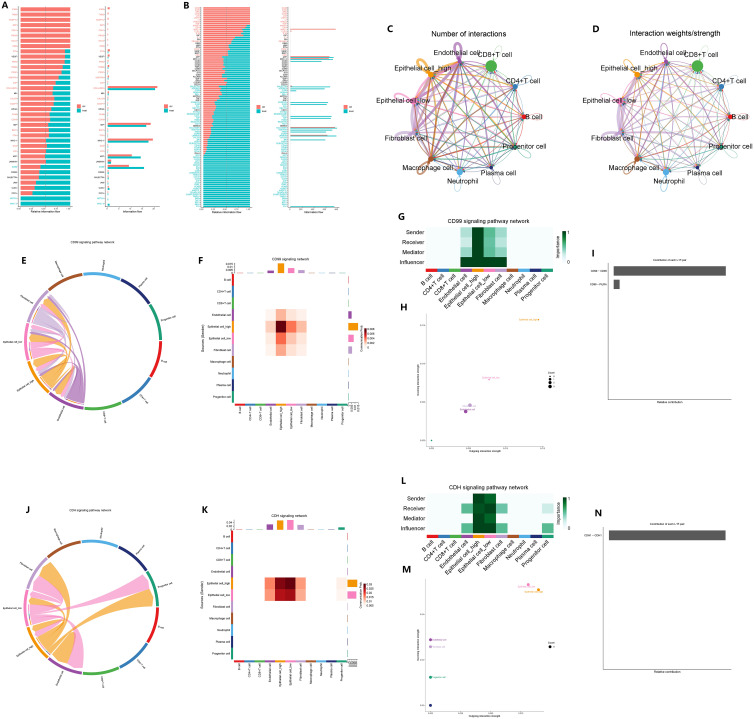
Effect of GADD45B on signal communication in gastric cancer microenvironment. **(A)** Microenvironmental signal changes in chronic atrophic gastritis pathogenesis. **(B)** Changes in microenvironment signals during the pathogenesis of gastric cancer. **(C)** Signaling pathways in which epithelial cells undergo significant changes during the pathogenesis of GC. **(C, D)** The importance of GADD45B-high gastric cancer cells and GADD45B-low gastric cancer cells in the communication network in the GC microenvironment. **(E–H)** The main participants of the CD99 signaling pathway network in the GC microenvironment and the importance of the participants. **(I)** CD99-CD99 is the most important ligand-receptor in the CD99 signaling pathway network. **(J–M)** The main participants of the CDH signaling pathway network in the GC microenvironment and the importance of the participants. **(N)** CDH1-CDH1 was the most important ligand-receptor in the CDH signaling pathway network.

We conducted a comparison of the signaling pathways between CAG and non-atrophic gastritis, as well as between GC and normal gastric tissue. Our analysis revealed that the CD99 signaling pathway was upregulated in the microenvironment of CAG, while the CDH signaling pathway was downregulated in the microenvironment of GC (**Figures 12A, B**). To further explore the impact of GADD45B on signal transduction in the microenvironment of GC tumors, we compared the cell communication between GADD45B-high and GADD45B-low GC cells. We found that GADD45B-high GC cells had closer communication with other cells in the tumor microenvironment than GADD45B-low GC cells, with vascular endothelial cells, tumor-associated fibroblasts, and tumor-associated macrophages serving as the primary signal-transmitting cells (**Figures 12C, D**). We also investigated the roles of GC cells in the CD99 and CDH signaling pathways. In the CD99 signaling pathway, GC cells, tumor-associated fibroblasts, and vascular endothelial cells were the primary signal-transducing cells (**Figures 12E, F**). Among them, GADD45B-high GC cells were the most important cells in this pathway, as they acted as the most crucial signal transmitters and receivers (**Figures 12G, H**). The CD99-CD99 receptor pair was the most significant receptor pair in this pathway (**Figure 12I**). In contrast, in the CDH signaling pathway, GC cells, tumor-associated fibroblasts, progenitor cells, and vascular endothelial cells were primarily involved in signal transduction (**Figures 12J, K**). GADD45B-low GC cells played the most crucial role as signal receivers in this pathway, while GADD45B-high GC cells were the primary signal senders (**Figures 12L, M**). The CDH1-CDH1 receptor pair was the most significant receptor pair in this pathway (**Figure 12N**).

### 3.11 scMetabolism revealed the metabolic changes mediated by GADD45B in two pathological processes

**Figure 13:**
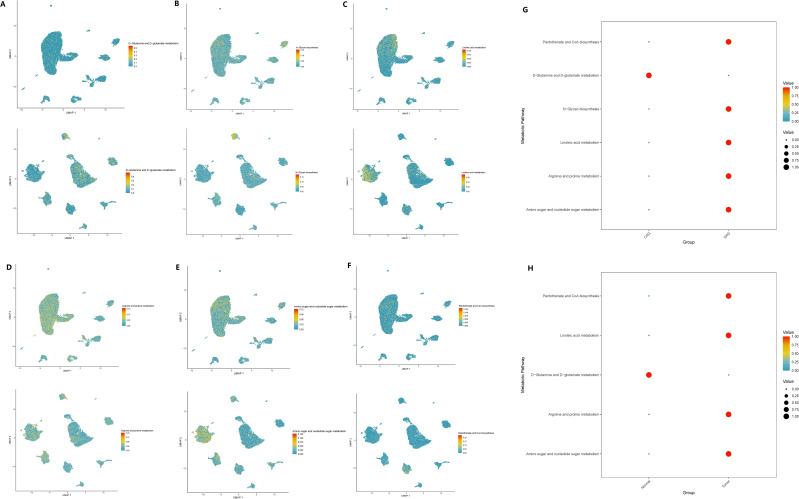
Expression changes of metabolic pathways in two diseases. **(A–F)** The enrichment of six key metabolic pathways in chronic atrophic gastritis and gastric cancer tissues. **(A)** D-glutamine and D-glutamate metabolism; **(B)** N-glycan biosynthesis. **(C)** Linoleic acid metabolism. **(D)** Arginine and proline metabolism; **(E)** Amino sugar and nucleotide sugar metabolism. **(F)** Pantothenate and CoA biosynthesis. **(G)** The expression levels of the six metabolic pathways in chronic atrophic gastritis and non-atrophic gastritis tissues. **(H)** The expression levels of the six metabolic pathways in gastric cancer and non-cancerous tissues.

Based on the scMetabolism package, we analyzed the results mapped to KEGG and observed significant alterations in metabolic pathways in GC cells overexpressing GADD45B. Three metabolic pathways were upregulated: Fructose and mannose metabolism (*P* = 0.003), D-Glutamine and D-glutamate metabolism (*P* = 0.007), and Amino sugar and nucleotide sugar metabolism (*P* = 0.002). Conversely, seven pathways were downregulated: Glycosylphosphatidylinositol (GPI)-anchor biosynthesis (*P* = 0.004), Neomycin, kanamycin, and gentamicin biosynthesis (*P* = 0.008), N-Glycan biosynthesis (*P* = 0.008), Linoleic acid metabolism (*P* = 0.023), Arginine and proline metabolism (*P* = 0.029), Ether lipid metabolism (*P* = 0.045), and Pantothenate and CoA biosynthesis (*P* = 0.049). We further investigated whether these ten metabolic pathways exhibited changes during the progression from CAG to gastric cancer. Our analysis revealed that eight metabolic pathways underwent significant changes during this transition. Notably, six pathways—D-Glutamine and D-glutamate metabolism (**Figure 13A**), N-Glycan biosynthesis (**Figure 13B**), Linoleic acid metabolism (**Figure 13C**), Arginine and proline metabolism (**Figure 13D**), Amino sugar and nucleotide sugar metabolism (**Figure 13E**), and Pantothenate and CoA biosynthesis (**Figure 13F**)—exhibited simultaneous alterations in both pathological processes. Among these pathways, D-Glutamine and D-glutamate metabolism, Linoleic acid metabolism, Arginine and proline metabolism, Amino sugar and nucleotide sugar metabolism, and Pantothenate and CoA biosynthesis displayed opposite trends in the two pathological processes. Conversely, N-Glycan biosynthesis exhibited consistent upregulation in both CAG and GC (**Figures 13G, H**).

### 3.12 Immunofluorescence and real-time quantitative PCR

**Figure 14:**
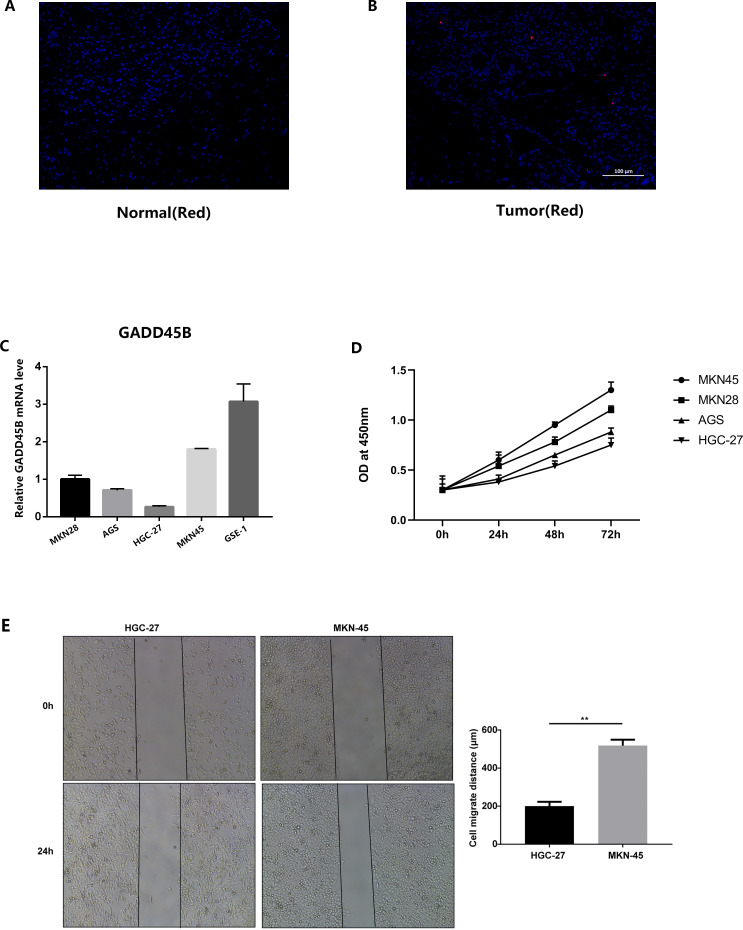
Results of *in vitro* experimental validation. **(A, B)** Immunofluorescence staining results of gastric cancer tissues and adjacent tissues. **(C)** The expression of GADD45B in normal gastric epithelial cell line (GES-1) and four gastric cancer cell lines (MKN28, AGS, HGC-27, MKN45). **(D)** CCK-8 experiments on four gastric cancer cell lines. **(E)** Analysis results of scratch experiments on MKN45 and HGC-27 cell lines. ** represent P < 0.01.

We conducted immunofluorescence staining using gastric cancer patients’ tissues and normal tissues obtained from our hospital. The findings indicated a noteworthy reduction in GADD45B expression in GC tissues compared to normal tissues (**Figures 14A, B**). Additionally, we performed PCR detection on GC cell lines and GC lines. The statistical analysis demonstrated that GADD45B expression was higher in the normal gastric epithelial cell line (GES-1) in comparison to GC cell lines (AGS, HGC-27, MKN28, and MKN45) (**Figure 14C**).

### 3.13 Cell function assay

The outcomes of the CCK-8 experiment revealed that GC cell lines exhibiting high levels of GADD45B expression displayed greater cell viability compared to those with low GADD45B expression (**Figure 14D**). This suggested that GADD45B might promote the growth of GC cell lines. In the scratch experiment, the experimental group (MKN45) exhibited a significant reduction in wound width and a shortened wound closure time compared to the control group (HGC-27). These observations indicated an increased migration speed of the cells (**Figure 14E**).

## 4 Discussion

Chronic atrophic gastritis, a recognized precursor to gastric cancer, is widely acknowledged to be the initial step in the cascade leading to gastric cancer. Numerous retrospective studies and evidence from basic research support the notion that CAG plays a pivotal role in the development of gastric cancer (61, 62). Consequently, transcriptomic alterations in atrophic gastritis may be closely linked to the generation of cancer cells. Previous investigations by various researchers have explored this association. For instance, Vytenis Petkevicius et al. examined the relationship between long-chain non-coding RNA ANRIL, H19, MALAT1, MEG3, HOTAIR polymorphisms, and gastric cancer and CAG. Their study demonstrated that lncRNAs influence CAG, leading to gastric cancer (63). Similarly, using high-throughput transcriptome data, Fan Zhang et al. identified CYP3A4 as a predictor for CAG progression and poor prognosis in gastric cancer (64). However, existing research on transcriptomic changes induced by CAG in gastric cancer still faces certain limitations. The scarcity of chronic CAG sequencing samples hampers extensive bioinformatics analyses, often limiting studies to a few bulk transcriptome datasets. Additionally, previous studies have not fully harnessed the latest bioinformatics and statistical methods to validate their findings. In this study, we aimed to investigate the transcriptome changes in gastric epithelial cells during the progression from CAG to gastric cancer using both bulk and single-cell transcriptome data. By identifying oncogenic drivers of CAG, we sought to explore their association with the immune microenvironment and the advancement of gastric cancer. Our objective was to offer a fresh perspective on the carcinogenic mechanisms underlying chronic atrophic gastritis and develop novel analytical approaches for bioinformatics research.

GADD45B is a member of the GADD45 gene family, which also includes GADD45A and GADD45G. It plays a crucial role in responding to physiological and environmental stresses, such as carcinogenic stress, by regulating cell cycle arrest, DNA repair, cell survival, senescence, and apoptosis (65). The expression of GADD45B is closely associated with the development and progression of various malignancies. Studies have demonstrated that low expression of GADD45B in normal tissues promotes carcinogenesis, whereas high expression in malignant tissues contributes to tumor development. In hepatocellular carcinoma, GADD45B expression is decreased in tumor tissues and different hepatocellular carcinoma cell lines, while it remains unchanged in normal liver tissues and cell lines. GADD45B acts as a critical regulator in the development of hepatocellular carcinoma (66). Patients with stage II colon cancer and high levels of GADD45B expression have shown poor overall survival (OS) and progression-free survival (PFS) rates (67). Elevated expression of GADD45B has also been identified as a prognostic factor for papillary thyroid cancer patients who have undergone complete thyroidectomy and radioactive iodine therapy, showing reduced disease-free survival (68). Additionally, GADD45B overexpression has been found to enhance the migration of ovarian cancer cells and is closely linked to ovarian cancer metastasis (69). Similar observations have been made in our research. We found that GADD45B plays distinct roles in different stages of gastric cancer. During the carcinogenic phase of chronic atrophic gastritis, GADD45B primarily inhibits gastric cancer development by suppressing the WNT signaling pathway. However, in advanced gastric cancer tissues, GADD45B exhibits a positive correlation with WNT signaling intensity, indicating its involvement in tumor progression. Furthermore, GADD45B promotes immunosuppression within the tumor microenvironment by modulating signaling interactions between gastric cancer cells and tumor-associated fibroblasts, vascular endothelial cells, and other microenvironmental cells. It also promotes the expression of immune checkpoints, thereby facilitating gastric cancer progression. Nevertheless, the precise mechanism underlying the functional switch of GADD45B remains to be elucidated, necessitating further research in this area.

Immune system is crucial for both immunotherapy and the growth of malignancies (12–14, 26, 70). Additionally, by analyzing the tumor immune microenvironment, it is possible to achieve predictions regarding the efficacy of tumor immunotherapy (71, 72). Our research focused on the role of GADD45B in regulating the immune microenvironment of GC patients. The expression level of GADD45B influenced various immune cells, including B cells, CD8+ T cells, monocytes, and others. It upregulated the expression of immune checkpoints, leading to immune suppression within the tumor microenvironment. Consequently, GC patients exhibited a highly immunosuppressive tumor microenvironment, which negatively impacted the effectiveness of chemotherapy and immunotherapy. Previous studies have indicated that GADD45B also controlled the functionality of immune cells. In malignant melanoma, GADD45B played a crucial role as an anti-tumor response participant by regulating the immune response of Th1 cells and CD8+ T cells (73). Additionally, GADD45B was found to modulate the innate immune function of granulocytes and macrophages by regulating p38 and JNK signaling pathways, which affected reactive oxygen species production, phagocytosis, adhesion, and chemotaxis (74). The immune regulation mediated by GADD45B in GC patients was a complex process. Consequently, targeting GADD45B has the potential to emerge as a novel therapeutic strategy for GC.

Multiple factors contribute to the continued progression of tumors in chemotherapy patients, including drug resistance (75–77). Chemotherapy is the primary treatment modality for advanced gastric cancer. Given the significant correlation between GADD45B and GC prognosis, GADD45B could serve as a predictive marker for the efficacy of pharmacological treatments in GC. Previous randomized controlled trials and basic experiments have shown the significant anti-gastric cancer activity of agents such as docetaxel (78), lapatinib (79), paclitaxel (80), methotrexate (81), gemcitabine (82), camptothecin (83), and cisplatin (84). Sensitivity analysis has revealed an inverse relationship between the expression level of GADD45B and the therapeutic response to docetaxel, lapatinib, and paclitaxel. Patients with low GADD45B expression might exhibit better treatment response, while those with high GADD45B expression might have a poorer response. Consequently, in clinical practice, low GADD45B expression could guide the selection of docetaxel, lapatinib, or paclitaxel as treatment options. Furthermore, immunotherapy has emerged as an innovative and promising approach for GC treatment (85). Consequently, our research predicted the impact of GADD45B on immunotherapy for patients with GC. In this context, our research explored the potential impact of GADD45B on immunotherapy for GC patients. We observed that GC tissues with high expression of GADD45B were characterized by a highly immunosuppressive microenvironment, suggesting that immunotherapy may have a more favorable effect in such cases. These findings hold significant clinical implications for the pharmacological management of GC.

The WNT signaling pathway plays a crucial role in various physiological processes, such as embryonic development, lineage commitment, adult stem cell homeostasis, and tissue regeneration (86). As one of the earliest signaling pathways identified in the tumor microenvironment, the WNT pathway has been implicated in carcinogenesis. The WNT family consists of 19 secreted glycoproteins, and its downstream effects could be categorized into β-catenin-dependent and β-catenin-independent pathways, depending on the requirement of β-catenin. Upon binding of WNT ligands, the co-receptors LRP5/6 and FZD are activated, leading to the recruitment of AXIN1/2 and DVL to the membrane (active WNT signals). This results in the destruction of the destruction complex, leading to the stabilization and nuclear translocation of β-catenin. In the nucleus, β-catenin interacts with TCF and LEF, recruiting coactivators such as p300 and CBP to promote the transcription of WNT target genes (87, 88). Previous studies have established a link between the WNT signaling pathway and the development of CAG and GC. Helicobacter pylori, a major pathogen associated with CAG and GC, could activate the WNT signaling pathway in gastric epithelial cells through various mechanisms. In gastric cancer, Helicobacter pylori upregulates the expression of Wnt10a and Wnt10b, thereby activating the Wnt/β-catenin pathway (89, 90). Additionally, helicobacter pylori infection in gastric epithelial cells leads to rapid activation of the co-receptor for the Wnt/β-catenin pathway, LRP6, resulting in the nuclear accumulation of β-catenin (91). Through GSVA analysis of single-cell transcriptome data, we identified the WNT signaling pathway as a co-expressed pathway in the pathogenesis of both CAG and gastric cancer. Furthermore, analysis of the TCGA cohort revealed a negative association between the WNT signaling pathway and GADD45B expression. GADD45B acts as a tumor suppressor during the progression from chronic atrophic gastritis to gastric cancer by inhibiting the WNT signaling pathway.

The expression level of GADD45B in GC cells has a significant impact on the disease’s progression and biological characteristics. GC cells with high GADD45B expression exhibit enhanced invasiveness and closer interactions with the immune microenvironment. Within the tumor microenvironment, GC cells with high GADD45B expression are prominently involved in the CD99 signaling network, while those with low GADD45B expression predominantly participate in the CDH signaling network. CD99 is a cell surface protein with diverse properties and an incompletely understood mechanism of action. It is involved in critical biological processes such as cell adhesion, migration, death, differentiation, and inflammation (92), as well as immune system regulation and cancer development. In numerous cancers, CD99 is highly expressed (93). In the CDH network, CDH1-CDH1 serves as the principal ligand receptor. CDH1 encodes cadherin-1, a calcium-dependent cell adhesion glycoprotein comprising five extracellular cadherin repeats, a transmembrane domain, and a conserved cytoplasmic tail. CDH1 is vital for maintaining cell adhesion and adhesion junctions in normal tissues (94). Reduced CDH1 expression or function in tumor tissues increases the likelihood of distant metastasis (95). According to our research, GC cells with high GADD45B expression play a crucial role in the CD99 and CDH1 networks. They primarily communicate with other tumor cells, fibroblasts, and vascular endothelial cells through CD99-CD99 interactions within the CD99 network. On the other hand, GC cells with low GADD45B expression participate in the primary CDH signaling pathway network and interact with other tumor cells, fibroblasts, and vascular endothelial cells via CDH1-CDH1 ligand receptors. Analysis of GC tissues from the TCGA cohort revealed a positive correlation between GADD45B and CD99 expression, while GADD45B exhibited a negative correlation with CDH1 expression. This suggests that GADD45B regulates the invasiveness of GC cells to promote disease progression. Moreover, GC cells with high GADD45B expression display greater invasiveness compared to those with low GADD45B expression. The tumor microenvironment in GC plays a pivotal role in disease progression, particularly during the advanced stage of invasion.

Since the discovery of the Warburg effect, it has become evident that metabolism plays a crucial role in diseases development (96). Advancements in high-throughput sequencing technology have enabled a more integrated analysis of transcriptomics and metabolomics, allowing for a comprehensive examination of metabolic pathways in GC at the single-cell level. In the pathogenesis of CAG, inflammation-induced damage to gastric mucosal epithelial cells leads to significant downregulation of most metabolic pathways. However, during the progression of gastric cancer, these downregulated metabolic pathways were reactivated and often surpass their original levels. Thus, the identification of six co-expressed metabolic pathways in our study hold great significance in understanding the progression of GC. Glutamine, a vital and widely utilized nutrient, played diverse roles in cancer cells, including energy formation, redox homeostasis, macromolecule synthesis, and signal transduction (97). N-glycan, a common protein modification in mammals, is closely associated with cancer growth, invasion, and metastasis (98). Linoleic acid, acting through various classical tumor pathways such as the Akt pathway, induces cancer cell invasion and migration (99). Amino sugar and nucleotide sugar metabolism are involved in the synthesis and glycosylation modification of molecules like proteins and lipids, exhibiting high activity in tumor cells (100). Pantothenic acid and coenzyme A have the capacity to regulate the anti-tumor immunity of different immune cells (101). In summary, GADD45B exerted a significant influence on the metabolic pathways of gastric cancer cells. Targeting GADD45B in treatment strategies might prove effective in restoring abnormal metabolic capacities within tumor tissues. The findings from our study provided valuable insights for the development of novel therapeutic approaches aimed at modulating gastric cancer metabolism.

Our study has certain limitations. Firstly, the carcinogenic mechanism of GADD45B regulation in chronic atrophic gastritis is a complex process, and this study only provides preliminary evidence. Further research can utilize more *in vitro* and *in vivo* models, as well as larger-scale clinical samples, to validate the role of GADD45B in carcinogenesis of chronic atrophic gastritis. Secondly, due to the lack of evidence, the application of our research findings in the clinical setting is challenging. Therefore, a significant amount of prospective research is still needed to validate our conclusions. Lastly, the absence of sequencing data may lead to inevitable biases, potentially compromising the reliability of the results. Hence, large-scale sequencing and integrative analysis are still necessary.

## 5 Conclusion

GADD45B, a cancer-related gene, has been implicated in the development of chronic atrophic gastritis. Low levels of GADD45B expression in gastric epithelial cells contribute to the activation of the WNT signaling pathway, thus promoting the carcinogenic process associated with chronic atrophic gastritis. Moreover, GADD45B plays a crucial role in mediating the communication between gastric cancer cells and the immune microenvironment, resulting in enhanced invasiveness and poor prognosis for GC patients. Additionally, GADD45B exerted broad regulatory effects on the metabolic pathways involved in chronic atrophic gastritis and gastric cancer tissues, ultimately facilitating the progression of gastric cancer.

## Data availability statement

The original contributions presented in the study are included in the article/**Supplementary Material**, further inquiries can be directed to the corresponding author/s.

## Ethics statement

The studies involving human participants were reviewed and approved by The Ethics Committee of the First Affiliated Hospital of Soochow University. The patients/participants provided their written informed consent to participate in this study.

## Author contributions

WX and YL conceived the study. WX, MZ, KS, DZ and CX drafted the manuscript. WX, TJ and DZ performed the literature search and collected the data. WX, MZ, TJ, and DZ analyzed and visualized the data. KS and DZ completed all experiments. WX, YL, WZ and CX helped with the final revision of this manuscript. All authors reviewed and approved the final manuscript.

## Supplementary material

Supplementary Table 1 Primer sequence of GADD45B.

Supplementary Table 2 Baseline data of gastric adenocarcinoma patients in the TCGA database.

Supplementary Figure 1 Changes in the number of cells in each group before and after quality control.

Supplementary Figure 2 The process and results of cell type annotation.
