# Supplementary material for: GADD45B regulates the carcinogenesis process of chronic atrophic gastritis and the metabolic pathways of gastric cancer
Source: Front Endocrinol (Lausanne). 2023 Aug 7;14:1224832. doi: 10.3389/fendo.2023.1224832 (PMC10441793; doi:10.3389/fendo.2023.1224832)
Supplement: Supplementary Table 1 — Primer sequence of GADD45B. [file Table_1.docx]

Forward primer ATCGCCCTGCAAATCCACTT

Reverse primer GTGTGAGGGTTCGTGACCAG
