# Supplementary material for: GADD45B regulates the carcinogenesis process of chronic atrophic gastritis and the metabolic pathways of gastric cancer
Source: Front Endocrinol (Lausanne). 2023 Aug 7;14:1224832. doi: 10.3389/fendo.2023.1224832 (PMC10441793; doi:10.3389/fendo.2023.1224832)
Supplement: Supplementary Table 2 — Baseline data of gastric adenocarcinoma patients in the TCGA database. [file Table_2.docx]

| Characteristics | Low expression of SOS2 | High expression of SOS2 | P value |
| --- | --- | --- | --- |
| n | 187 | 188 |  |
| Pathologic T stage, n (%) |  |  | 0.019 |
| T1&T2 | 54 (14.7%) | 45 (12.3%) |  |
| T3 | 94 (25.6%) | 74 (20.2%) |  |
| T4 | 39 (10.6%) | 61 (16.6%) |  |
| Pathologic N stage, n (%) |  |  | 0.666 |
| N0 | 62 (17.4%) | 49 (13.7%) |  |
| N1 | 48 (13.4%) | 49 (13.7%) |  |
| N2 | 36 (10.1%) | 39 (10.9%) |  |
| N3 | 36 (10.1%) | 38 (10.6%) |  |
| Gender, n (%) |  |  | 0.859 |
| Female | 66 (17.6%) | 68 (18.1%) |  |
| Male | 121 (32.3%) | 120 (32%) |  |
| Race, n (%) |  |  | 0.094 |
| Asian&Black or African American | 49 (15.2%) | 36 (11.1%) |  |
| White | 112 (34.7%) | 126 (39%) |  |
| Pathologic M stage, n (%) |  |  | 0.802 |
| M0 | 163 (45.9%) | 167 (47%) |  |
| M1 | 13 (3.7%) | 12 (3.4%) |  |
| Age, n (%) |  |  | 0.024 |
| <= 65 | 93 (25.1%) | 71 (19.1%) |  |
| > 65 | 93 (25.1%) | 114 (30.7%) |  |
| OS event, n (%) |  |  | 0.363 |
| Alive | 118 (31.5%) | 110 (29.3%) |  |
| Dead | 69 (18.4%) | 78 (20.8%) |  |
| Pathologic stage, n (%) |  |  | 0.133 |
| Stage I | 28 (8%) | 25 (7.1%) |  |
| Stage II | 67 (19%) | 44 (12.5%) |  |
| Stage III | 69 (19.6%) | 81 (23%) |  |
| Stage IV | 18 (5.1%) | 20 (5.7%) |  |
| Histologic grade, n (%) |  |  | 0.274 |
| G1 | 3 (0.8%) | 7 (1.9%) |  |
| G2 | 74 (20.2%) | 63 (17.2%) |  |
| G3 | 107 (29.2%) | 112 (30.6%) |  |
