# Supplementary figures and images for: GADD45B regulates the carcinogenesis process of chronic atrophic gastritis and the metabolic pathways of gastric cancer
Source: Front Endocrinol (Lausanne). 2023 Aug 7;14:1224832. doi: 10.3389/fendo.2023.1224832 (PMC10441793; doi:10.3389/fendo.2023.1224832)

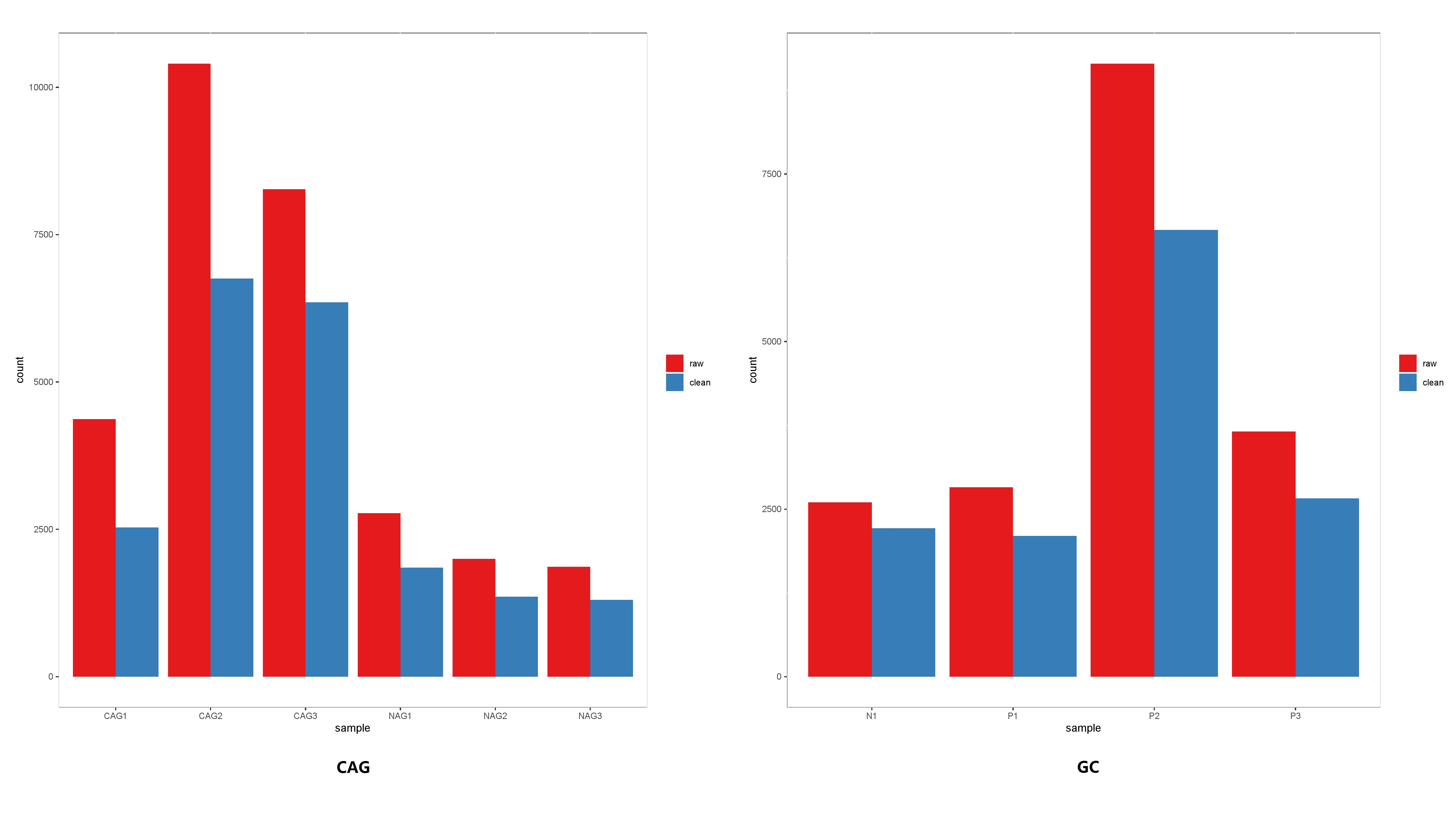

Supplement: Supplementary Figure 1 — Changes in the number of cells in each group before and after quality control. [file Image_1.tif]
