# Supplementary material for: GADD45B regulates the carcinogenesis process of chronic atrophic gastritis and the metabolic pathways of gastric cancer
Source: Front Endocrinol (Lausanne). 2023 Aug 7;14:1224832. doi: 10.3389/fendo.2023.1224832 (PMC10441793; doi:10.3389/fendo.2023.1224832)

1. Single cell annotation of chronic Atrophic gastritis matrix

(1) B cell

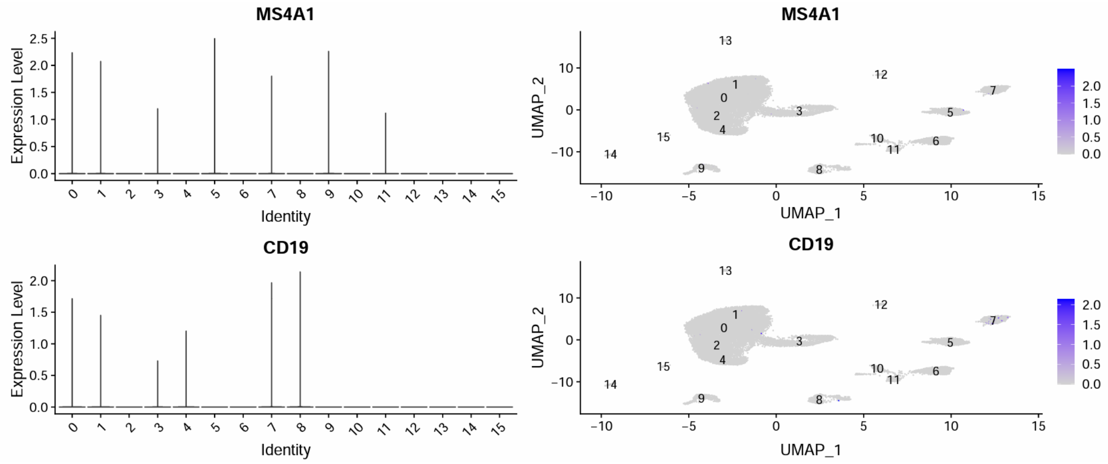

(2) CMP

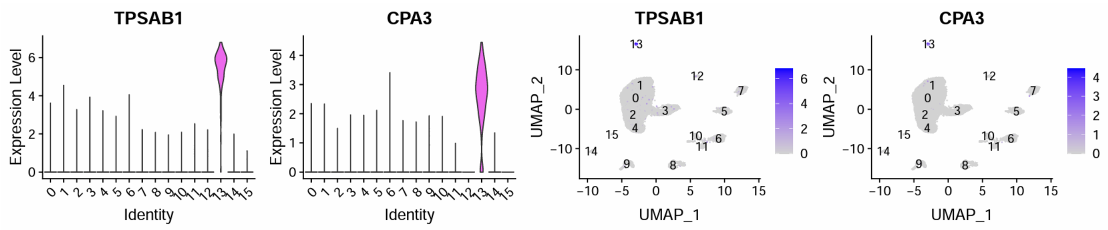

(3) Endothelial cell

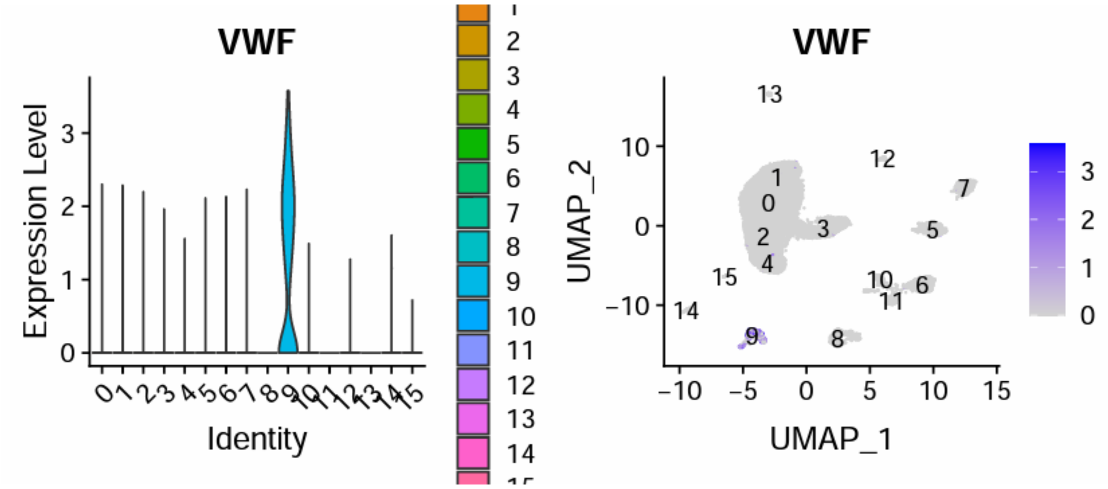

(4) Epithelial cell

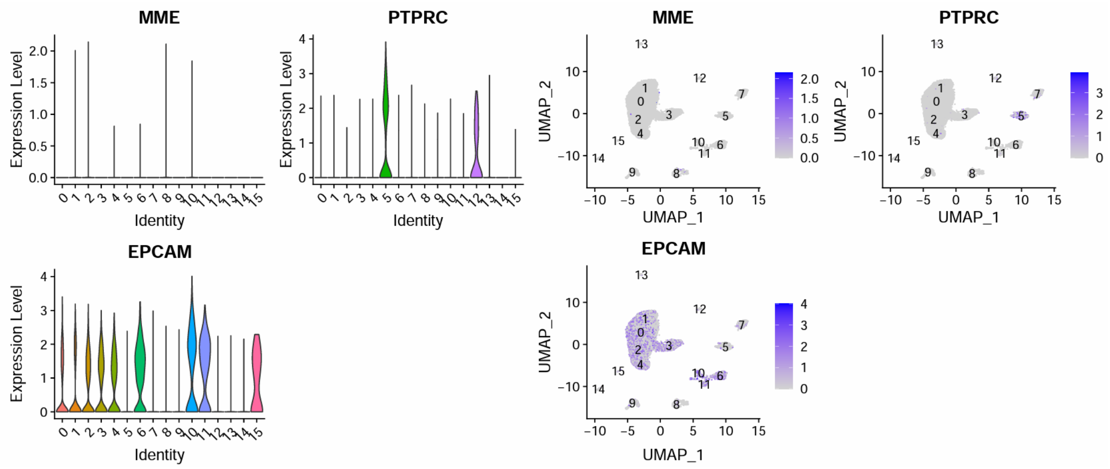

(5) Fibroblast

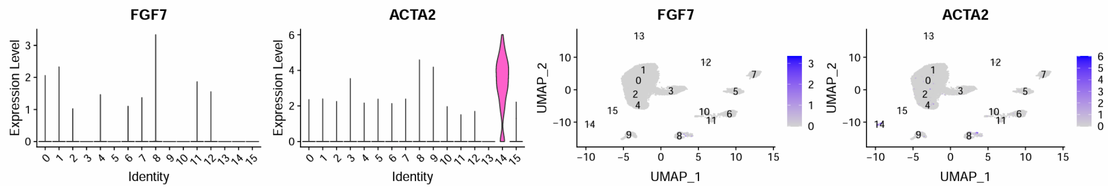

(6) Macrophage

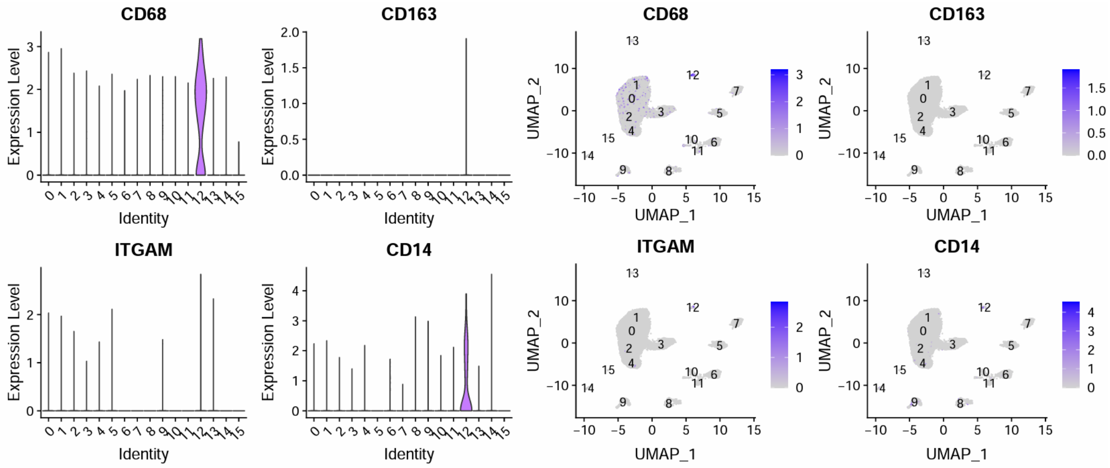

(7) CD8+T cell

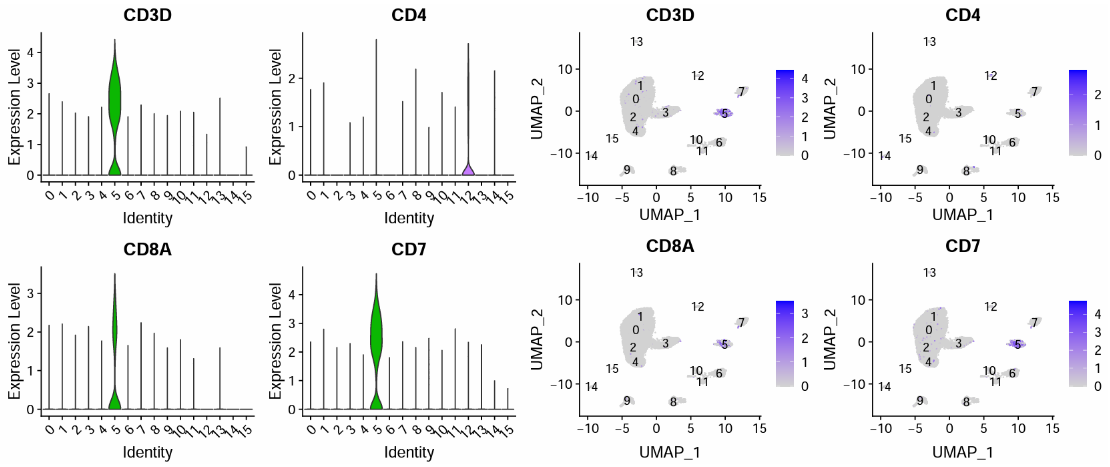

(8) TSC

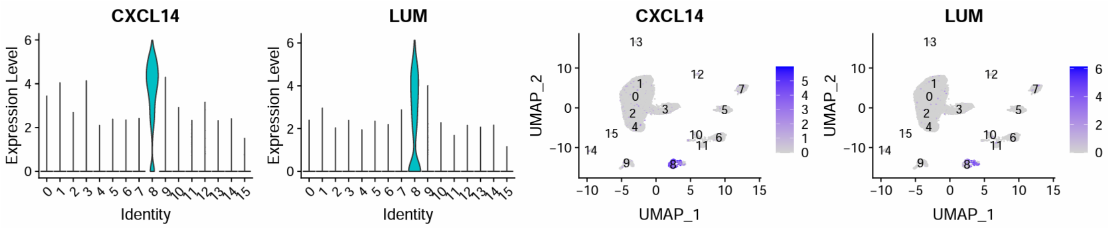

2. Single cell annotation of gastric cancer matrix

(1) B cell

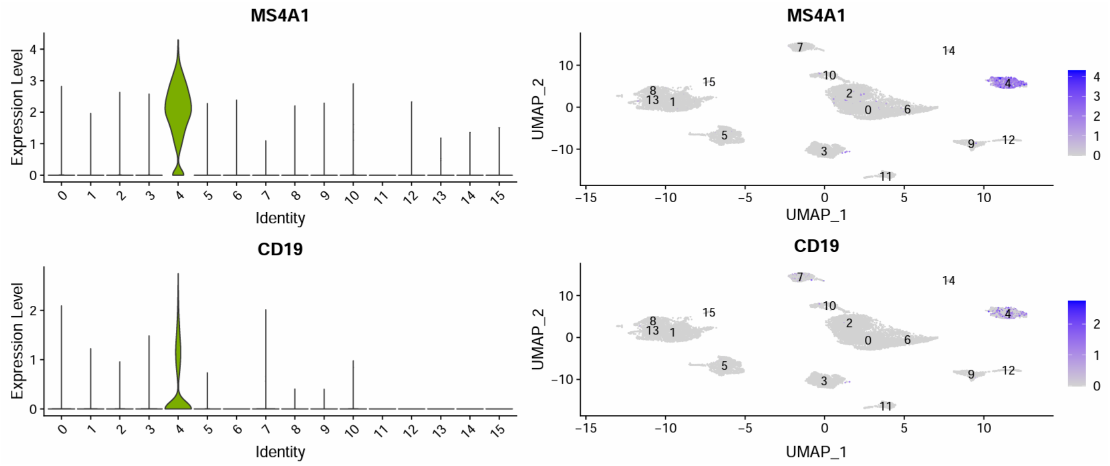

(2) CMP

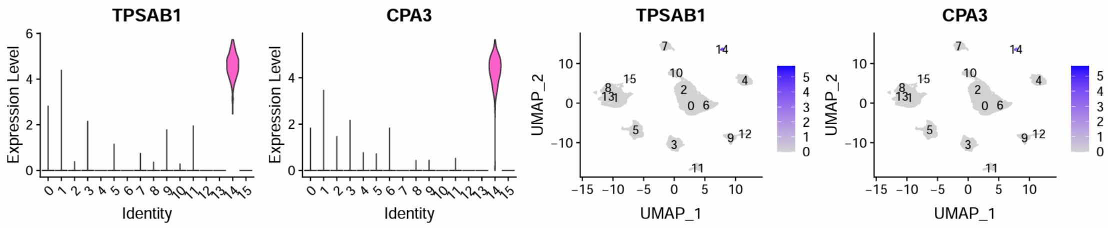

(3) Endothelial cell

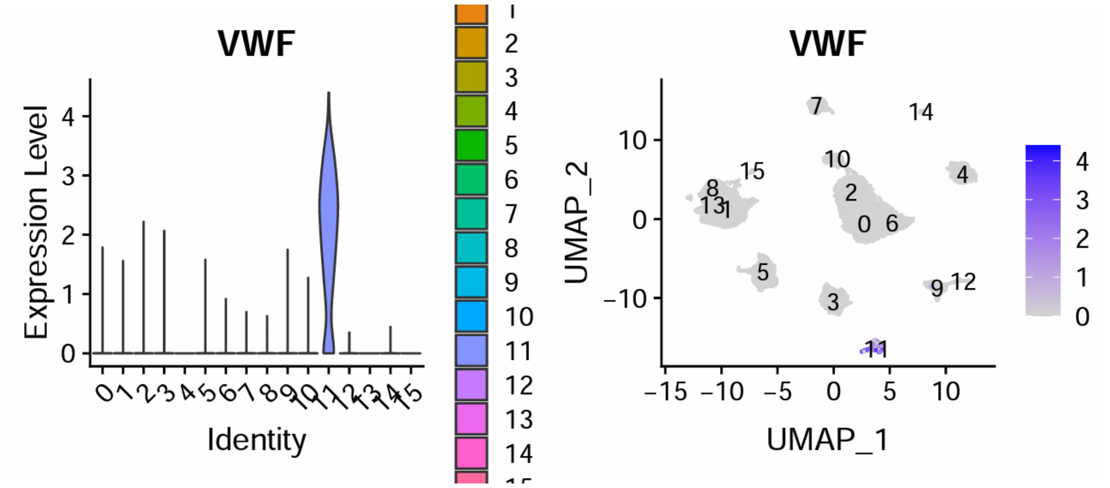

(4) Fibroblast

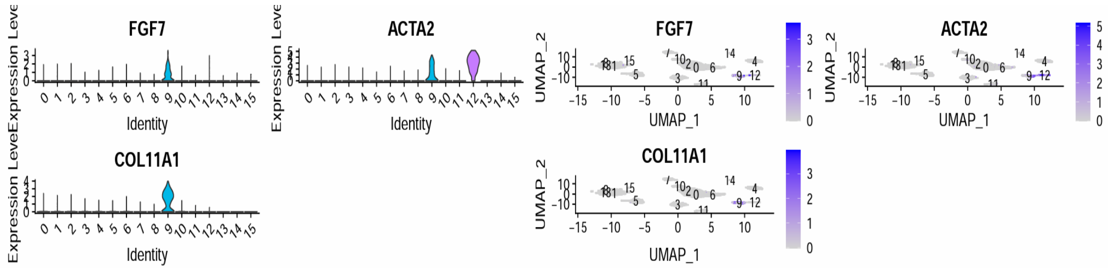

(5) Macrophage

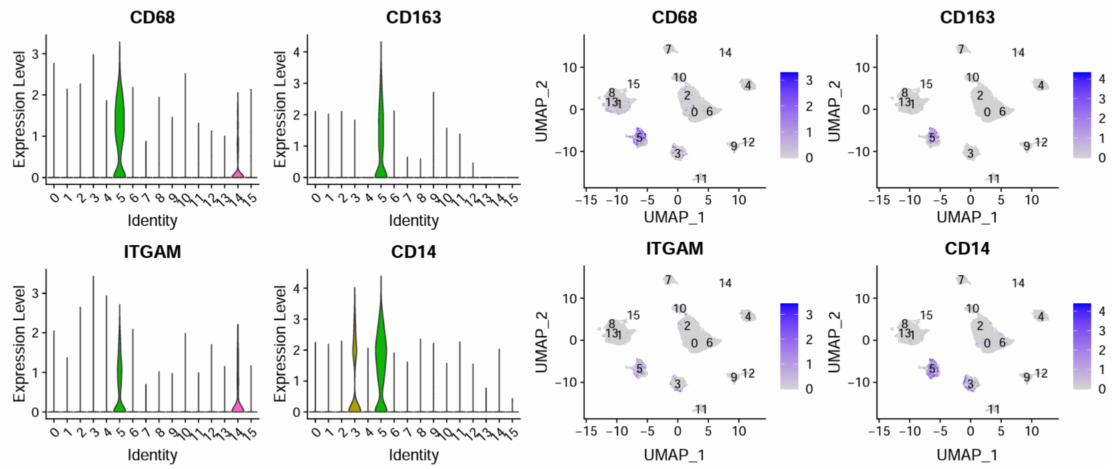

(6) Neutrophil

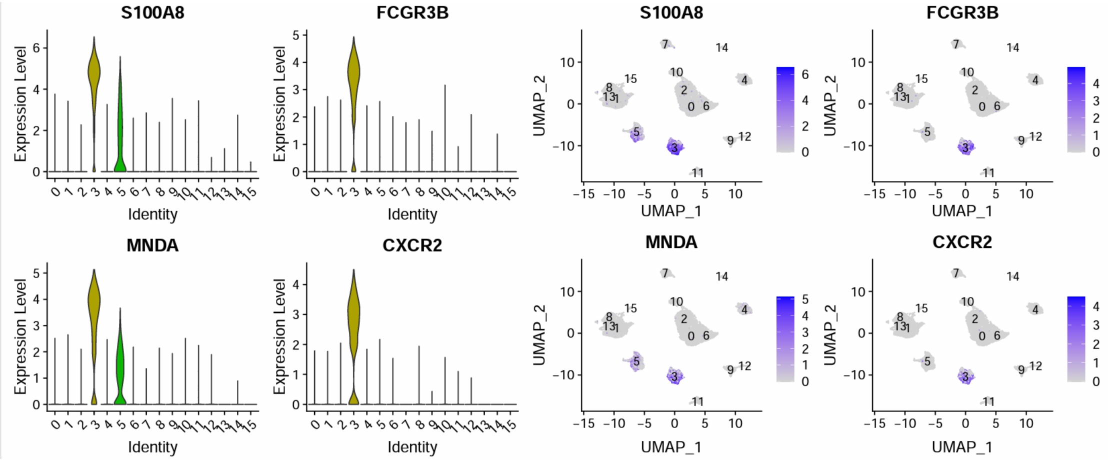

(7) Plasma

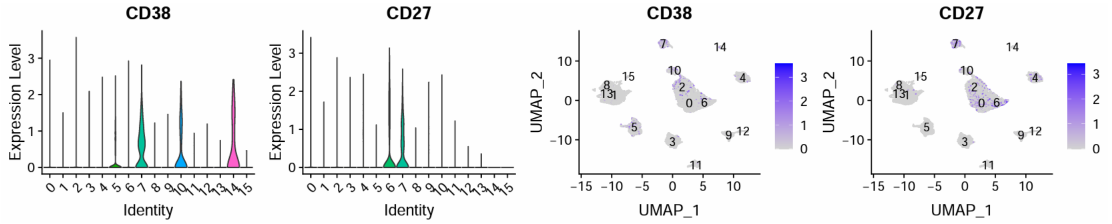

(8) Progenitor

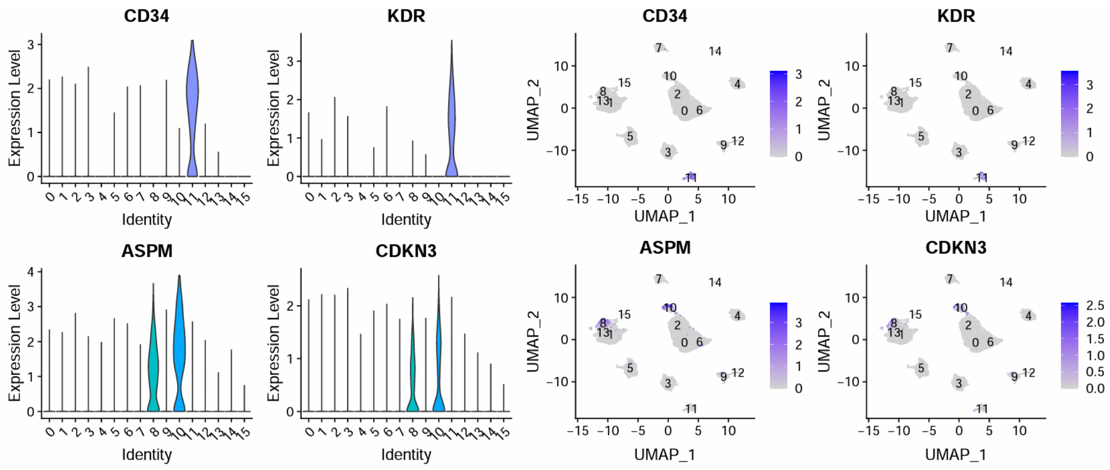

(9) CD4+T cell and CD8+T cell

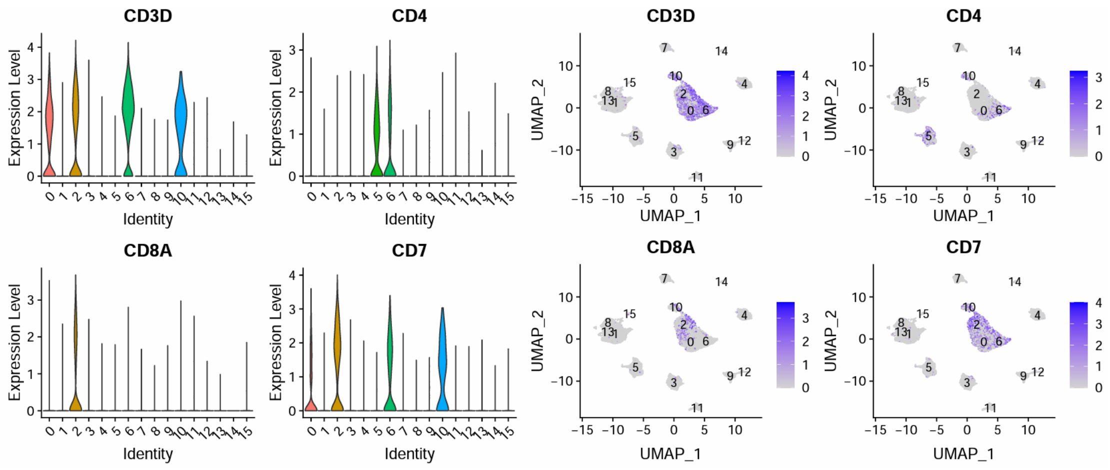

(10) Epithelial

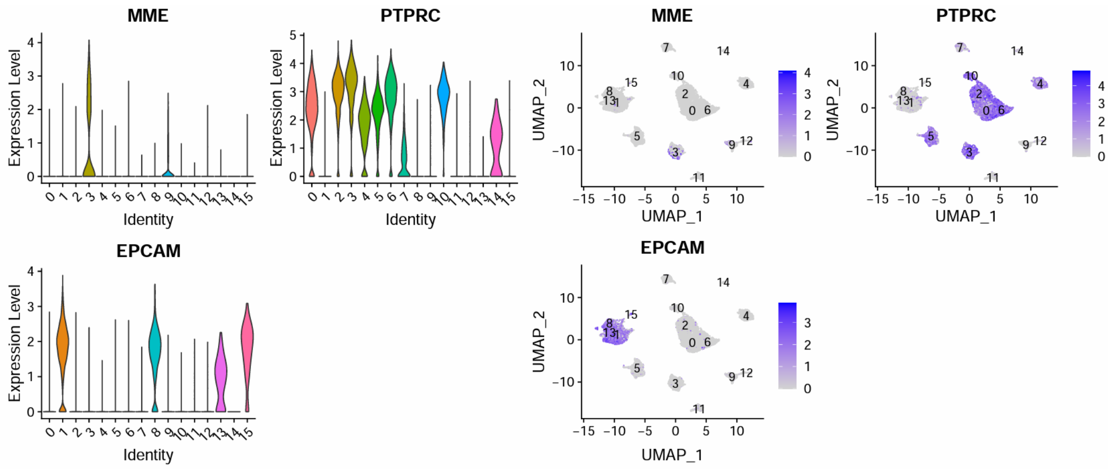

Supplement: Supplementary Figure 2 — The process and results of cell type annotation. [file Image_2.pdf]
